# Inflammation, metabolism, and aging in heart failure with preserved ejection fraction: Mechanisms and treatment perspectives

**DOI:** 10.1515/jtim-2026-0043

**Published:** 2026-06-13

**Authors:** Yaqun Teng, Weikang Bian, Qingyu Li, Jennifer E Van Eyk, Xiaowei Yan, Shuyang Zhang

**Affiliations:** Department of Cardiology, State Key Laboratory of Complex Severe and Rare disease, Peking Union Medical College Hospital, Chinese Academy of Medical Sciences and Peking Union Medical College, Beijing, China; Department of Internal Medicine, Peking Union Medical College Hospital, Chinese Academy of Medical Sciences and Peking Union Medical College, Beijing, China; Advanced Clinical Biosystems Research Institute, Smidt Heart Institute, Cedars-Sinai Medical Center, Los Angeles, CA, USA; School of Medicine, Tsinghua Medicine, Tsinghua University, Beijing, China

**Keywords:** heart failure with preserved ejection fraction, microvascular inflammation, metabolic remodeling, aging, translational medicine

## Abstract

Heart failure with preserved ejection fraction (HFpEF) is an increasingly prevalent clinical syndrome with limited effective therapies, representing a major unmet need in cardiovascular medicine. A comorbidity-driven systemic proinflammatory state, together with coronary microvascular endothelial inflammation, has emerged as a central paradigm in HFpEF pathogenesis. Accumulating evidence over the past decade has highlighted the extensive crosstalk between inflammation, metabolic dysregulation, and aging-related processes. In this review, we summarize key advances defining the roles of systemic and microvascular inflammation, metabolic abnormalities, and cellular senescence, and integrate these findings into an interconnected inflammation–metabolism–aging axis in HFpEF. We further discuss the current clinical and emerging preclinical therapeutic strategies targeting these pathways. By linking mechanistic insights with translational perspectives, this review provides a conceptual framework to guide precise therapeutic development for this complex and heterogeneous syndrome.

## Introduction

Heart failure (HF) is a clinical syndrome characterized by structural and/or functional cardiac abnormalities, elevated B-type natriuretic peptide levels, and pulmonary or systemic congestion, representing an advanced stage of diverse cardiac diseases. Heart failure with preserved ejection fraction (HFpEF) is defined by a left ventricular ejection fraction of ≥ 50% accompanied by elevated filling pressures.^[1]^ The prevalence of HF continues to increase, with HFpEF now accounting for approximately half of all cases and occurring more commonly in women.^[2,3]^ Despite its increasing burden, HFpEF is associated with poor prognosis, with 5-year mortality rates of 50%–75%.^[4]^ Effective therapies for heart failure with reduced ejection fraction (HFrEF) largely fail in HFpEF, and no treatment has yet demonstrated a significant mortality benefit in this population;^[5]^ consequently, HFpEF represents a primary unmet clinical need in cardiology.

HFpEF is a clinically heterogeneous syndrome driven by multiple cardiovascular and metabolic risk factors, including obesity, diabetes, hypertension, chronic kidney disease, and aging. It encompasses several overlapping phenotypic subgroups, such as the metabolic‑obese phenotype, older individuals with vascular aging, and relatively younger individuals with low B-type natriuretic peptide levels, among others.^[6,7]^ Key pathophysiologic processes in HFpEF include left ventricular diastolic dysfunction, left atrial cardiomyopathy, atrial fibrillation, pulmonary hypertension, right ventricular dysfunction, disturbances in autonomic and adrenergic signaling, skeletal muscle dysfunction, and adipose tissue overload.^[8]^ Notably, rare etiologies such as amyloidosis and hereditary cardiomyopathies are distinct from the more common cardiometabolic forms of HFpEF and are beyond the scope of this review.

Understanding the molecular pathogenesis of HFpEF is essential for the development of effective therapies. Over the past decade, substantial progress has been made in elucidating key mechanisms, particularly systemic low-grade chronic inflammation and its crosstalk with cardiac metabolism and aging, thereby markedly advancing our understanding of HFpEF biology. To integrate these advances, we performed a systematic literature review. A targeted search of PubMed (National Library of Medicine) was conducted using the core term “HFpEF” in combination with “inflammation”, “metabolism”, or “aging”. Retrieved articles were critically appraised, and the principal pathophysiological pathways and emerging therapeutic strategies associated with this axis were identified and synthesized. This primary search was augmented by screening reference lists of seminal reviews and original studies to ensure comprehensive incorporation of both established and recent insights. In this article, we first delineate the roles of systemic and coronary microvascular inflammation, metabolic dysregulation, and aging-related mechanisms in the pathogenesis of HFpEF. We then integrate these components to illustrate their dynamic crosstalk and convergent pathogenic pathways. Finally, we review current and emerging therapeutic strategies that target these interconnected mechanisms. By coupling mechanistic insights with therapeutic developments, this review aims to emphasize the translational potential of HFpEF research.

## Paradigm of microvascular inflammation

HFpEF was traditionally regarded as a consequence of hypertensive afterload leading to myocardial hypertrophy and diastolic dysfunction. In 2013, Paulus and Tschöpe proposed a paradigm in which a comorbidity-driven systemic proinflammatory state induces coronary microvascular endothelial inflammation, thereby playing a central role in HFpEF pathogenesis.^[9]^ This inflammatory process promotes myocardial stiffness and fibrosis and exerts extracardiac effects on the lungs, kidneys, skeletal muscle, adipose tissue, vasculature, and immune system.^[9, 10, 11]^ Unlike the secondary inflammatory response that follows cardiomyocyte loss in HFrEF, systemic inflammation in HFpEF is considered a primary, causal contributor involving multiple interconnected pathophysiologic processes.^[12]^

**Figure 1:**
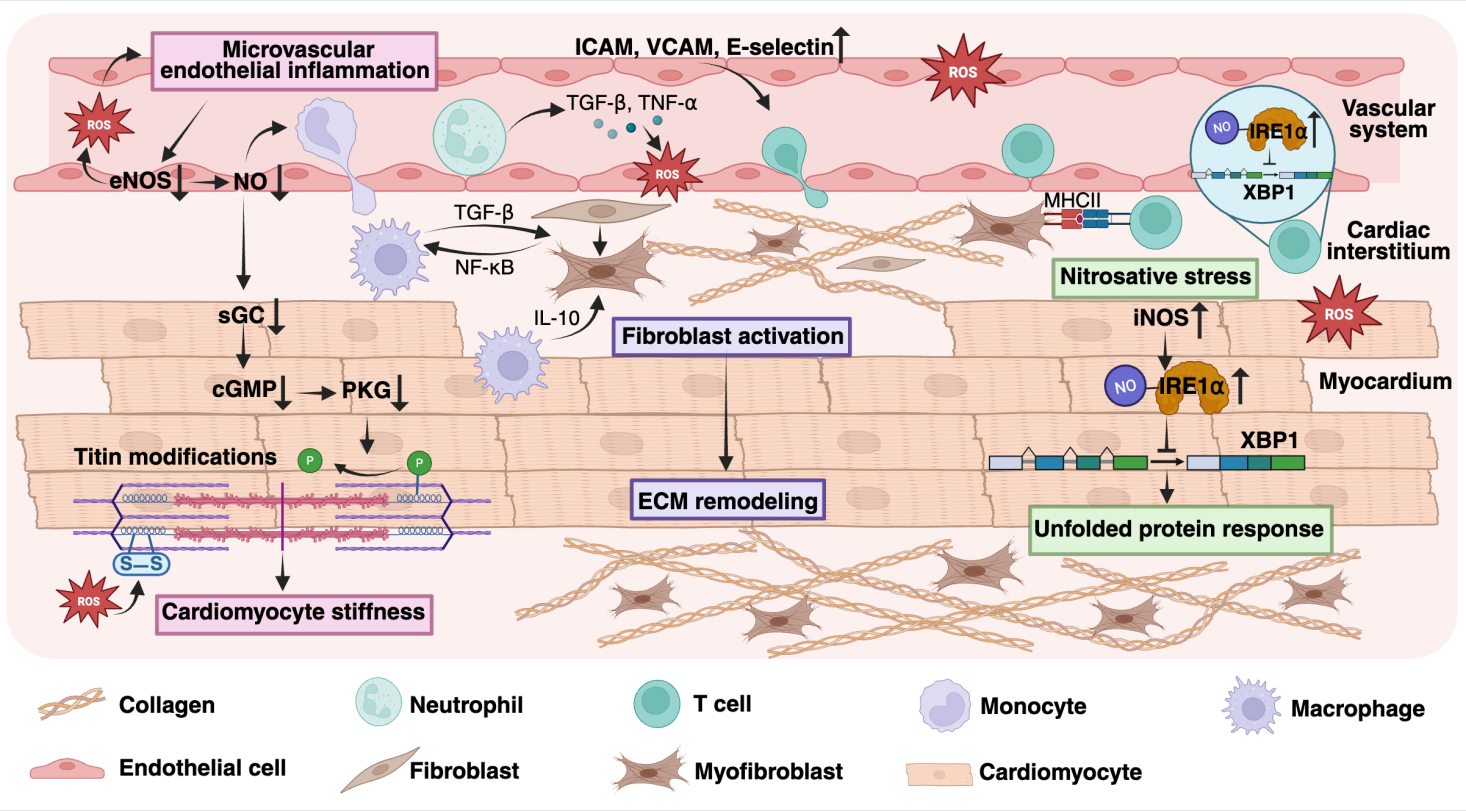
Paradigm of microvascular inflammation in HFpEF. A comorbidity-driven systemic proinflammatory state induces coronary microvascular endothelial inflammation, which impairs NO–cGMP–PKG signaling, reduces titin N2B phosphorylation, and increases cardiomyocyte stiffness. In parallel, microvascular inflammation recruits macrophages, activates fibroblasts, and promotes ECM remodeling. Oxidative and nitrosative stress further trigger iNOS-mediated S-nitrosylation of IRE1α and aberrant XBP1 signaling, disrupting protein homeostasis and modulating T cell infiltration. Together, these mechanisms contribute to diastolic dysfunction in HFpEF. cGMP: cyclic guanosine monophosphate; ECM: extracellular matrix; eNOS: endothelial nitric oxide synthase; HFpEF: heart failure with preserved ejection fraction; iNOS: inducible nitric oxide synthase; IRE1α: inositol-requiring enzyme 1α; NO: nitric oxide; PKG: protein kinase G; ROS: reactive oxygen species; sGC: soluble guanylate cyclase; XBP1: X-box-binding protein 1; ICAM: intercellular adhesion molecule; VCAM: vascular cell adhesion molecule; TGF-β: transforming growth factor-β; TNF-α: tumor necrosis factor-α; NF-κB: nuclear factor kappa-B.

The HFpEF paradigm encompasses several key mechanistic steps and has evolved with accumulating evidence (Figure 1): (1) Comorbidities such as obesity, diabetes, hypertension, chronic kidney disease, and chronic obstructive pulmonary disease trigger chronic systemic inflammation. (2) This systemic proinflammatory state promotes coronary microvascular endothelial inflammation. (3) The inflamed endothelium generates reactive oxygen species (ROS), reduces endothelial nitric oxide synthase activity, and limits nitric oxide (NO) diffusion into cardiomyocytes, thereby attenuating cyclic guanosine monophosphate (cGMP) –protein kinase G (PKG) signaling. (4) Reduced PKG activity promotes cardiomyocyte hypertrophy and increases passive tension through hypophosphorylation of the titin N2B domain. (5) Elevated oxidative and nitrosative stress, driven by proinflammatory cytokines, enhances inducible nitric oxide synthase (iNOS) activity and induces pathologic S-nitrosylation of inositol-requiring enzyme 1α (IRE1α), disrupting the unfolded protein response and impairing protein quality control. (6) In parallel, inflammation induces myocardial interstitial fibrosis and extracellular matrix remodeling *via* transforming growth factor beta (TGF-β) signaling and immune–fibroblast crosstalk. (7) The combined effects of increased cardiomyocyte stiffness and interstitial fibrosis culminate in diastolic dysfunction and overt HF.

Beyond this endothelial-centered framework, cardiomyocyte-intrinsic mechanisms, including mitochondrial dysfunction, metabolic derangement, DNA damage responses, and cellular senescence, among others, also contribute to HFpEF pathogenesis. These mechanisms intersect with inflammation, metabolism, and aging, reflecting the integrated nature of the disease process and the evolving complexity of the HFpEF model. The following sections will examine the evidence supporting each step of this paradigm, as well as the emerging complementary pathways.

### Evidence of systemic and myocardial inflammation in HFpEF

HFpEF is characterized by chronic, low-grade systemic inflammation, primarily driven by metabolic comorbidities and aging. This state is evidenced by elevated circulating inflammatory biomarkers and myocardial infiltration by immune cells.^[13]^ Increased circulating levels of C-reactive protein, interleukins (IL-6, IL-1β, IL-10), tumor necrosis factor (TNF), myeloperoxidase, monocyte chemoattractant protein-1, soluble ST2, growth differentiation factor-15 (GDF-15), and pentraxin-3 have been consistently reported in patients with HFpEF.^[13, 14, 15]^ These biomarkers correlate with disease activity and adverse prognosis.^[16, 17, 18]^ In addition, network analyses of large HF cohorts have identified HFpEF-specific enrichment of inflammatory and extracellular matrix remodeling pathways,^[19]^ highlighting the central role of inflammation in HFpEF pathophysiology.

Myocardial tissue from patients with HFpEF shows increased infiltration of monocytes, macrophages, neutrophils, and T cells.^[15,20]^ This immune cell recruitment is driven by endothelial activation and upregulation of adhesion molecules, including intercellular adhesion molecule-1 (ICAM-1) and E-selectin, along with an imbalance in immune cell subsets, characterized by increased T helper 17 (Th17) cells and reduced regulatory T cells.^[21,22]^ Single-cell RNA sequencing studies in HFpEF mouse models have further demonstrated expansion of proinflammatory macrophage populations that actively drive disease pathogenesis.^[23,24]^ Cardiac inflammation and oxidative stress are tightly coupled and form a self-perpetuating vicious cycle.^[22,25]^ Inflammatory mediators such as TNF and TGF-β impair mitochondrial function and promote excessive ROS generation.^[22,25]^ In turn, ROS induce oxidative DNA damage and activate poly (ADP-ribose) polymerase 1, which triggers the nuclear factor kappa-light-chain-enhancer of activated B cells (NF-κB) and NLR family pyrin domain-containing 3 (NLRP3) inflammasome pathways, thereby sustaining chronic inflammation and promoting adverse cardiac remodeling.^[25,26]^

### Coronary microvascular endothelial inflammation in HFpEF

Coronary microvascular dysfunction (CMD) is a prevalent feature of HFpEF and was identified in approximately 75% of patients in the PROMIS-HFpEF study, as reflected by reduced coronary flow reserve and elevated biomarkers of endothelial dysfunction.^[27]^ CMD is closely linked to common cardiometabolic comorbidities and is associated with subclinical myocardial injury, diastolic dysfunction, and an increased risk of major adverse cardiovascular events, providing a shared pathogenic basis for both HFpEF and microvascular angina.^[28,29]^ Beyond endothelial-dependent CMD, endothelial-independent impairments in microvascular reactivity show a strong association with mortality in HFpEF,^[30]^ emphasizing the heterogeneity of CMD in this condition.

Mechanistically, vascular endothelial inflammation represents a major driver of CMD.^[28]^ Transcriptomic analyses of myocardial tissue from patients with HFpEF demonstrate upregulation of adhesion molecules, including vascular cell adhesion molecule 1, ICAM- 1, and E-selectin, which facilitate leukocyte adhesion, transmigration, and activation.^[21,31]^ These inflammatory processes promote endothelial dysfunction, impair NO–cGMP–PKG signaling, and disrupt cardiomyocyte relaxation. In mouse models, ICAM-1 deficiency attenuates monocyte and T cell infiltration, reduces cardiac fibrosis, and is associated with lower IL-6 and IL-1β expression, supporting a causal role for endothelial inflammation in CMD and fibrotic remodeling.^[32]^

Another important contributor to CMD is coronary microvascular rarefaction, which reflects structural loss of capillaries and impaired angiogenesis.^[28]^ Transcriptomic profiling of myocardial tissue from patients with HFpEF reveals downregulation of angiogenic pathways.^[33]^ Diabetes mellitus further exacerbates CMD through systemic microvascular injury, myocardial capillary rarefaction, and impaired angiogenesis, thereby contributing to the development and progression of HFpEF.^[34]^ Consistent with these findings, single-cell transcriptomic analyses of HFpEF myocardium demonstrate reduced endothelial cell populations characterized by pro-apoptotic and anti-angiogenic gene signatures.^[35]^ Notably, fibroblasts in HFpEF hearts overexpress angiopoietin-like 4, which inhibits endothelial angiogenesis and promotes collagen deposition, highlighting a pathologic fibroblast–endothelial cell interaction that exacerbates both microvascular rarefaction and fibrosis.^[35]^

### Role of the NO-cGMP-PKG pathway and titin modifications

The NO–cGMP–PKG signaling pathway plays a central role in the regulation of vascular tone and endothelial function and exerts critical effects on cardiomyocyte physiology.^[13,36]^ NO, synthesized by endothelial nitric oxide synthase in vascular endothelial cells, diffuses into adjacent vascular smooth muscle cells and cardiomyocytes, where it activates soluble guanylate cyclase (sGC). This activation leads to cGMP production, which, in turn, activates PKG. Under physiologic conditions, this pathway maintains cardiovascular homeostasis by exerting antihypertrophic, antifibrotic, and proangiogenic effects, thereby preventing maladaptive cardiac remodeling.^[36]^ In pathological states such as inflammation, coronary microvascular endothelial dysfunction is characterized by increased nitrosative and oxidative stress, resulting in reduced expression and activity of endothelial nitric oxide synthase. This leads to diminished NO production and bioavailability, accompanied by increased ROS generation.^[37]^ Both clinical studies and preclinical HFpEF models consistently demonstrate reduced NO and sGC bioavailability, decreased cGMP levels, and impaired PKG activity.^[21,38]^ In this setting, reduced PKG activity not only reduces titin phosphorylation, thereby increasing cardiomyocyte stiffness, but also contributes to inflammation, hypertrophy, and fibrosis, all of which drive the progression of HFpEF. ^[10,36]^

Titin, a giant sarcomeric protein that is essential for myocardial passive tension, is tightly regulated by the NO–cGMP–PKG pathway and by multiple post-translational modifications that modulate its elastic properties.^[39]^ In patients with HFpEF, titin shows hypophosphorylation of the N2B domain and hyperphosphorylation of the PEVK domain, both of which increase myocardial passive tension.^[40,41]^ A shift toward the stiffer N2B isoform relative to the more compliant N2BA isoform has been observed in animal models of HFpEF,^[42]^ although this change has not been consistently demonstrated in human studies.^[41]^ Oxidative stress further increases cardiac stiffness in part through redox modifications of titin. *In vivo* studies show that oxidation of the distal spring elements of titin, *via* mechanisms such as disulfide cross-linking and unfolded-domain oxidation, alters titin elasticity, promotes domain aggregation, and enhances phosphorylation, collectively contributing to increased passive tension and diastolic dysfunction.^[43]^

### Nitrosative stress and the unfolded protein response in myocardial dysfunction

Beyond the protective role of NO signaling in endothelial cells, systemic inflammation increases iNOS expression in cardiomyocytes *via* NF-κB activation. This leads to the production of high concentrations of NO, which directly suppresses sGC activity. In parallel, excess NO rapidly reacts with superoxide anions to form cytotoxic peroxynitrite, inducing nitrosative stress and further exacerbating diastolic dysfunction.^[44]^ Inhibition or genetic deletion of iNOS prevents obesity-induced CMD in mouse models,^[45]^ highlighting the critical role of iNOS-mediated nitrosative stress in endothelial-independent CMD and HFpEF.

The unfolded protein response, particularly the IRE1α–X-box-binding protein 1 (XBP1) axis, is downregulated in a double-hit HFpEF mouse model combining a high-fat diet with *N*^ω^-nitro-L-arginine methyl ester treatment, implicating impaired protein quality control in cardiometabolic HFpEF.^[46]^ Mechanistically, systemic inflammation upregulates cardiomyocyte iNOS expression, promoting pathologic S-nitrosylation of IRE1α and abnormal XBP1 splicing, which leads to protein accumulation and myocardial dysfunction. Consistent with this mechanism, pharmacologic inhibition or genetic ablation of iNOS in mice mitigates mitochondrial dysfunction, oxidative stress, and Akt S-nitrosylation in cardiomyocytes, resulting in improvement of the HFpEF phenotype.^[47]^ Notably, dysregulation of the IRE1α–XBP1 axis also occurs in T cells in HFpEF mouse models, where it facilitates myocardial T cell infiltration and contributes to cardiac inflammation.^[48]^

### Mechanisms of inflammation-induced fibroblast activation and myocardial interstitial fibrosis

Systemic inflammation promotes myocardial interstitial fibrosis and extracellular matrix (ECM) remodeling, ultimately contributing to diastolic dysfunction.^[22,28]^ Increased expression of adhesion molecules on vascular endothelial cells facilitates monocyte recruitment and differentiation into macrophages, which secrete TGF-β and drive fibrotic remodeling.^[21,37,49]^ TGF-β induces fibroblast-to-myofibroblast differentiation, enhances collagen synthesis, suppresses matrix metalloproteinase activity, and increases tissue inhibitors of metalloproteinases.^[50,51]^ In parallel, reduced NO bioavailability promotes immune cell adhesion and directly accelerates fibroblast activation.^[28]^ In addition, NO depletion, oxidative stress, and TGF-β signaling act synergistically to induce endothelial-to-mesenchymal transition, further expanding the fibroblast pool and amplifying myocardial fibrosis.^[28,52]^

Immune cell–fibroblast interactions further promote fibroblast activation and myofibroblast differentiation. Cardiac macrophages secrete IL-10, which activates fibroblasts, promotes collagen deposition, and contributes to diastolic dysfunction.^[53]^ Other immune cells, including dendritic cells, T and B lymphocytes, as well as proinflammatory cytokines such as IL-6, IL-1, and IL-18, also stimulate fibroblast activation.^[22]^ Activation of NF-κB signaling in fibroblasts promotes monocyte recruitment to the myocardium.^[54]^ Moreover, fibroblasts can function as antigen-presenting cells by upregulating major histocompatibility complex class II expression in response to interferon-γ, thereby activating CD4^+^ T cells and further exacerbating myocardial inflammation and dysfunction.^[55]^ These reciprocal interactions underscore the intricate immunofibrotic network that characterizes HFpEF.

Although inflammation is a major driver of fibrosis in HFpEF, emerging transcriptomic data demonstrate profibrotic gene signatures even in the absence of active inflammatory programs, suggesting that ECM remodeling can occur independently of overt inflammation.^[56]^ Epigenetic mechanisms may also contribute directly. For example, inhibition of histone deacetylases (HDACs) suppresses fibroblast activation by disrupting chromatin recruitment of the profibrotic reader bromodomain-containing protein 4.^[57]^ Furthermore, overexpression of GATA4 in cardiac fibroblasts reduces fibrosis and improves diastolic function by suppressing fibroblast activation without inducing cardiomyocyte transdifferentiation, highlighting a potential antifibrotic therapeutic strategy.^[58]^

## Cardiac energy metabolism abnormalities

### Metabolic inflexibility

Dysregulated myocardial energy metabolism is a key contributor to the development of HF.^[59,60]^ Under physiologic conditions, fatty acid β-oxidation supplies approximately 60%–90% of myocardial adenosine triphosphate (ATP), with glucose, ketone bodies, lactate, and other substrates providing complementary contributions.^[60]^ In HF, the myocardium becomes energetically compromised and loses metabolic flexibility, defined as the capacity to switch adaptively among energy substrates in response to physiologic or pathologic demands.^[11,59,61]^ Whereas HFrEF is often described as “an engine out of fuel, ” cardiometabolic HFpEF is characterized by fatty acid overload and a forced reliance on fatty acid oxidation, without the compensatory shift toward glucose and ketone utilization typically observed in HFrEF.^[62,63]^ This distinct metabolic phenotype has led to the emerging concept of “energy resilience” to describe the unique energetic impairments in HFpEF.^[62]^

Transcriptomic analyses of human myocardium demonstrate upregulation of mitochondrial ATP synthesis and electron transport pathways in HFpEF compared with control tissue, in contrast to their downregulation in HFrEF.^[33]^ However, myocardial proteomic studies in patients with severe obesity and HFpEF reveal impaired fatty acid uptake, processing, and oxidation, accompanied by marked lipid droplet accumulation.^[64]^ Consistent with these findings, metabolomic analyses of HFpEF myocardium show reduced levels of fatty acid metabolism products, tricarboxylic acid cycle intermediates, ketone bodies, and branched-chain amino acid metabolites.^[65]^ Despite increased expression of glucose transporter type 1, levels of glycolytic and ancillary pathway intermediates, as well as expression of their associated enzymes, are also reduced in HFpEF.^[66]^ Together, these data indicate that impaired substrate oxidation and utilization underlie metabolic inflexibility and disrupted energy resilience in HFpEF pathogenesis.

### Cardiac metabolic remodeling across different comorbidities

**Figure 2:**
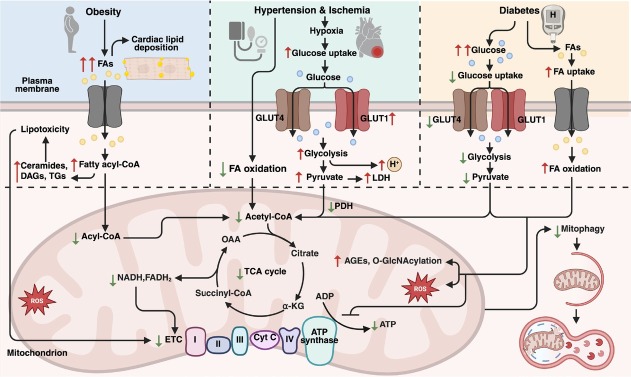
Metabolic derangements in HFpEF driven by comorbidities. Metabolic comorbidities, including obesity, hypertension, and diabetes, disrupt cardiac substrate utilization and mitochondrial function, leading to metabolic inflexibility in HFpEF. Obesity promotes excess fatty acid accumulation and generation of lipotoxic intermediates that impair mitochondrial ATP production. Hypertensive or ischemic states reduce fatty acid oxidation and uncouple glucose uptake from glucose oxidation, limiting energy generation. In diabetes, suppressed glucose utilization and increased fatty acid oxidation enhance glucotoxicity, oxidative stress, and mitochondrial injury. Impaired mitochondrial quality control through defective mitophagy further contributes to HFpEF pathogenesis. AGE: advanced glycation end product; ATP: adenosine triphosphate; DAG: diacylglycerol; ETC: electron transport chain; FA: fatty acid; FADH2: reduced flavin adenine dinucleotide; HFpEF: heart failure with preserved ejection fraction; LDH: lactate dehydrogenase; NADH: reduced nicotinamide adenine dinucleotide; OAA: oxaloacetate; PDH: pyruvate dehydrogenase; TG: triacylglycerol.

Distinct metabolic comorbidities drive specific patterns of cardiac metabolic remodeling. In obesity, elevated circulating free fatty acids lead to excessive myocardial lipid accumulation, including toxic lipids such as diacylglycerols, ceramides, and triglycerides. This lipid overload induces lipotoxicity, mitochondrial dysfunction, and impaired ATP production.^[61,64,67]^ In pressure overload or ischemic HF, fatty acid oxidation is disrupted, and hypoxia-driven increases in glucose uptake and glycolysis become uncoupled from efficient glucose oxidation, resulting in insufficient energy generation.^[59,60,68]^ In diabetic cardiomyopathy, both glucose oxidation and glycolysis are reduced, whereas aberrant fatty acid oxidation is increased, promoting glucotoxicity through advanced glycation end-products and O-linked β-N-acetylglucosamine modification, mitochondrial dysfunction, oxidative stress, and activation of cell death pathways.^[59,60,69]^ Together, these distinct metabolic remodeling processes contribute to the complexity and heterogeneity of metabolic dysregulation observed in HFpEF (Figure 2).^[61,65]^

### Mitochondrial dysfunction in HFpEF

Mitochondrial dysfunction represents a key abnormality in cardiac metabolism in HFpEF and is characterized by reduced ATP production, increased ROS generation, and structural mitochondrial damage.^[70,71]^ Ultrastructural analyses of myocardial tissue from patients with HFpEF reveal mitochondrial swelling with cristae separation and dissolution, particularly in individuals with severe obesity.^[64]^ Importantly, the HFpEF myocardium shows impaired activation of mitophagy, a critical mechanism of mitochondrial quality control.^[72]^ Accumulation of damaged mitochondria and release of cytosolic mitochondrial DNA (mtDNA) can activate cyclic GMP–AMP synthase–stimulator of interferon genes (cGAS-STING) signaling, thereby promoting cardiac inflammation and hypertrophy.^[73]^ Conversely, stimulation of fatty acid oxidation has been shown to enhance mitophagy, preserve mitochondrial integrity, and confer protection against HFpEF.^[72]^

## Cardiac aging and cellular senescence

### Mechanisms of aging and cellular senescence in HFpEF

**Figure 3:**
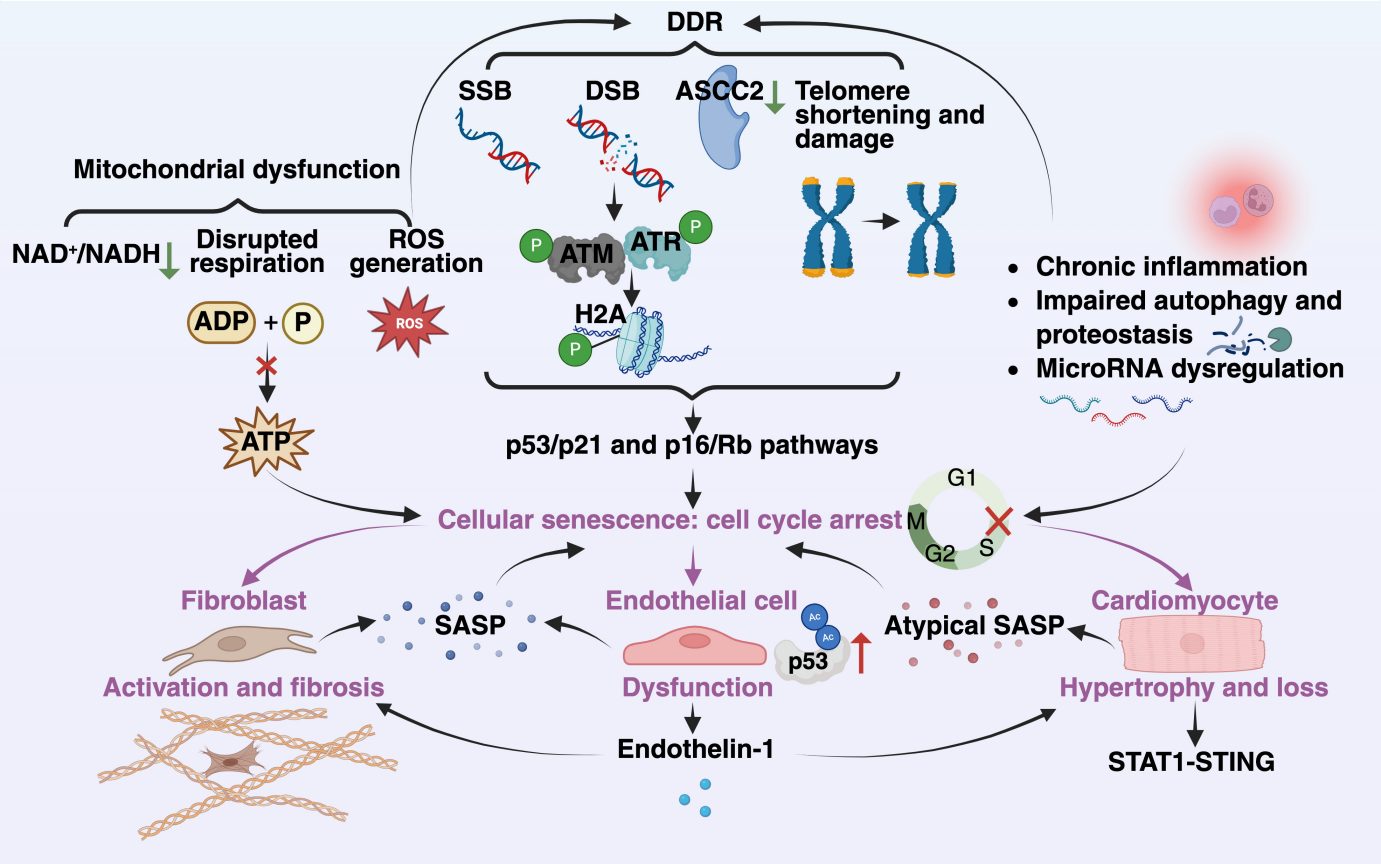
Cellular senescence in HFpEF. Cellular senescence in HFpEF arises from DNA damage, mitochondrial dysfunction, chronic inflammation, impaired autophagy and proteostasis, and dysregulated microRNA expression. Oxidative stress-induced DNA damage and telomere attrition activate ATM and ATR signaling and the p53/p21 and p16/Rb pathways, driving irreversible cell cycle arrest. Senescent cells exhibit mitochondrial dysfunction characterized by reduced NAD+ metabolism, impaired respiration, and excess ROS production. Endothelial senescence increases endothelin-1 expression, activating fibroblasts and promoting cardiomyocyte hypertrophy, whereas cardiomyocyte senescence involves STAT1–STING pathway activation. Senescent cardiac cells develop an SASP that propagates senescence through paracrine signaling, collectively promoting endothelial dysfunction, fibrosis, and hypertrophy in HFpEF. ASCC2: activating signal co-integrator 1 complex subunit 2; ATM: ataxia telangiectasia mutated; ATR: ataxia telangiectasia and Rad3-related; DDR: DNA damage response; DSB: double-strand break; HFpEF: heart failure with preserved ejection fraction; NAD+: nicotinamide adenine dinucleotide; NADH: reduced nicotinamide adenine dinucleotide; ROS: reactive oxygen species; SASP: senescence-associated secretory phenotype; SSB: single-strand break; STAT1: signal transducer and activator of transcription 1; STING: stimulator of interferon genes.

Aging is a major risk factor for incident HFpEF, with risk increasing by approximately 90% for every 10-year increase in age.^[74]^ Although the incidence of both HFpEF and HFrEF rises with advancing age, aging exerts a more pronounced effect on HFpEF.^[74,75]^ Cellular senescence, defined by irreversible cell cycle arrest associated with aging, is closely linked to cardiovascular diseases, including HF (Figure 3).^[76,77]^ Senescence can be induced by multiple stimuli, such as ROS, which activate the DNA damage response (DDR) and lead to permanent cell cycle arrest.^[76,78]^ Senescent cells acquire a senescence-associated secretory phenotype (SASP), characterized by the release of soluble signaling molecules, proteases, and insoluble protein and ECM components. This phenotype promotes a chronic inflammatory state and induces senescence in neighboring cells.^[78]^

Senescence across multiple cardiac cell types contributes to HFpEF pathogenesis. Endothelial cell senescence plays a central role in microvascular dysfunction. In a mouse model combining accelerated senescence with a high-fat diet, increased levels of acetylated p53, a marker of endothelial senescence, were associated with endothelial inflammation and diastolic dysfunction.^[31]^ Senescent endothelial cells also upregulate endothelin-1, which promotes fibroblast senescence and collagen production while inducing cardiomyocyte hypertrophy through paracrine signaling.^[28,79]^ In cardiomyocytes, exposure to interferon gamma induces senescence markers, including p53, p21, and γH2AX, along with activation of the signal transducer and activator of transcription 1 (STAT1) – STING pathway. Notably, these effects can be reversed by the sodium–glucose cotransporter 2 (SGLT2) inhibitor empagliflozin.^[80]^ Although cellular senescence clearly contributes to HFpEF, further studies are needed to delineate the distinct mechanisms by which senescence in specific cell types drives disease progression.

### Role of DDR in cellular senescence and HF

Inflammation and oxidative stress are key drivers of aging-related diseases, and DDR plays a critical role in oxidative stress-induced cellular senescence. Excessive ROS generated in the mitochondria and nucleus causes oxidative damage to nuclear DNA, including single-strand breaks (SSBs) and double-strand breaks (DSBs). This damage activates DDR signaling cascades, including ataxia telangiectasia and Rad3-related and ataxia telangiectasia mutated (ATM) kinases, increased γH2AX phosphorylation, and engagement of the p53/p21 and p16/Rb pathways, leading to cell cycle arrest and promotion of cellular senescence or apoptosis.^[76]^

Substantial evidence supports a central role for DDR in HF pathogenesis. In pressure overload-induced HF, ROS accumulation and SSBs occur in cardiomyocyte nuclei, a process exacerbated by deletion of the SSB repair protein, X-ray repair cross-complementing protein 1. Persistent unrepaired SSBs sustain DDR activation and upregulate inflammatory cytokine expression through NF-κB signaling, thereby worsening HF.^[81]^ Markers of DSBs, including γH2AX and phosphorylated ATM, are also elevated in this setting. Inhibition or cardiomyocyte-specific deletion of ATM attenuates cardiac hypertrophy and ventricular remodeling, indicating that excessive DDR activation directly contributes to disease progression.^[82]^ Consistent with these findings, increased phosphorylated ATM levels have also been observed in HFpEF mouse models.^[83]^ The DNA repair-associated factor activating signal co-integrator 1 complex subunit 2 (ASCC2) has emerged as a key protective regulator in HFpEF, with loss of its expression inducing HFpEF phenotypes in mice, accompanied by DDR activation and increased expression of proinflammatory cytokines such as IL-6 and TNF.^[83]^

Telomere shortening is a fundamental driver of cellular aging.^[84]^ With each cell division, telomeres progressively erode until reaching a critical length at which the shelterin complex can no longer protect the telomeric loop structure, triggering DDR activation and cell cycle arrest. In human hearts, cardiomyocyte telomere shortening represents a distinct hallmark of HF.^[85]^ Importantly, telomere dysfunction can also occur independently of telomere shortening. Telomeric DNA damage alone is sufficient to induce cardiomyocyte senescence, activate p21 and p16 signaling pathways, and promote the secretion of noncanonical SASP factors, including GDF-15, TGF-β2, and endothelin-3. These factors collectively contribute to myocardial fibrosis and hypertrophy.^[86]^

## Crosstalk among inflammation, metabolism, and aging

**Figure 4:**
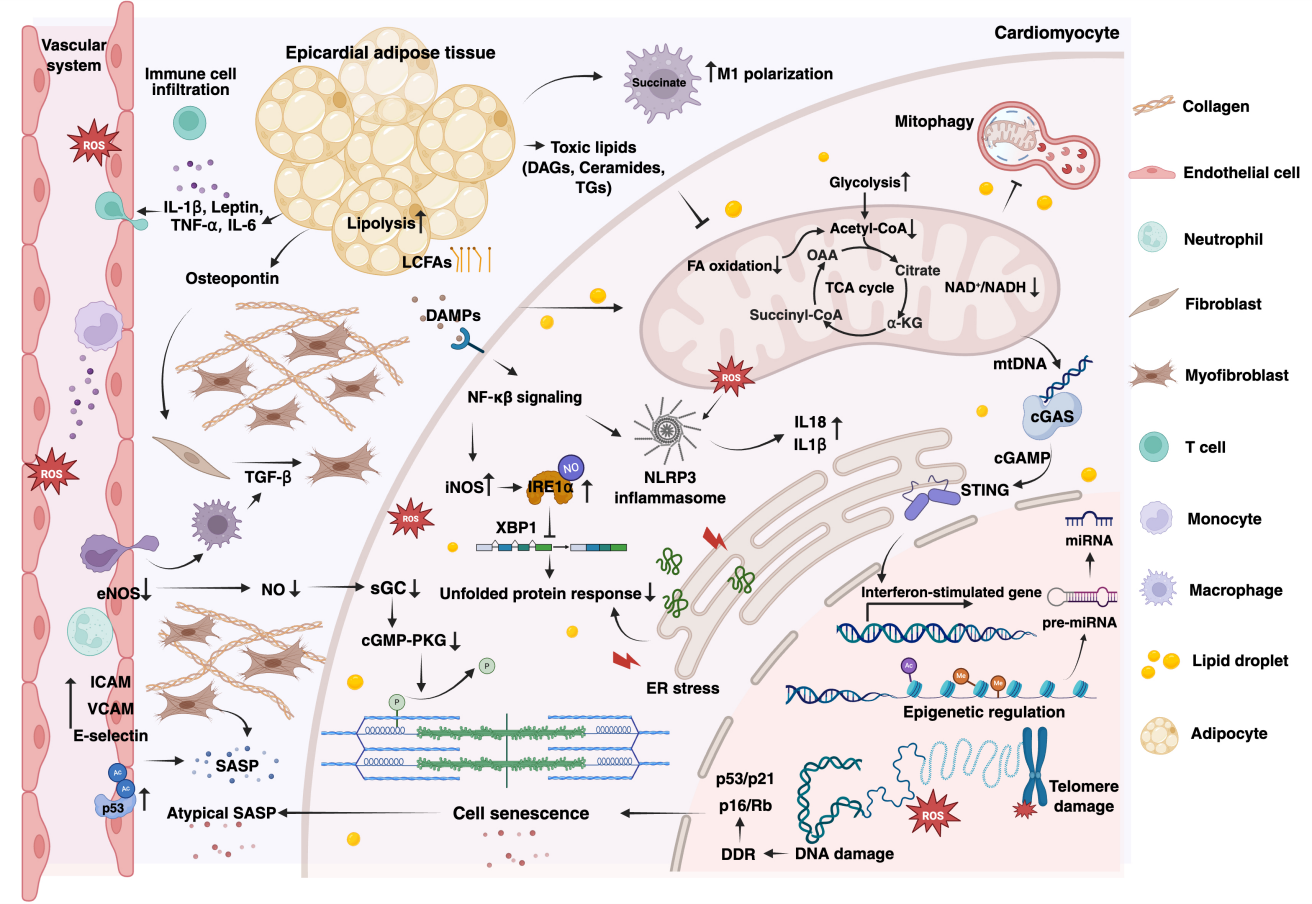
Integrated molecular pathways of the inflammation–metabolism–aging axis in HFpEF. EAT releases chemokines that promote immune cell infiltration, macrophage M1 polarization, and myocardial lipotoxicity. Activated macrophages secrete TGF-β, driving fibroblast-to-myofibroblast transdifferentiation, excessive collagen deposition, and myocardial fibrosis. Systemic inflammation induces coronary microvascular endothelial inflammation, leading to impaired NO–cGMP– PKG signaling, reduced titin N2B phosphorylation, and increased cardiomyocyte passive stiffness. In parallel, nitrosative stress disrupts the IRE1α–XBP1 axis, leading to dysfunction of UPR. DAMPs engage PRRs and activate NF-κB signaling, upregulating NLRP3 and pro-IL-1β/IL-18 transcription. Upon stimulation by mitochondrial ROS, the NLRP3 inflammasome assembles and becomes activated, resulting in the maturation and release of IL-1β and IL-18. Mitochondrial dysfunction in HFpEF is characterized by reduced FAO, a metabolic shift toward anaerobic glycolysis, NAD^+^ depletion, and impaired mitophagy. mtDNA released into the cytosol activates the cGAS–STING pathway, enhancing interferon-stimulated gene transcription. Excessive ROS further induce oxidative DNA damage, telomere damage, and DDR activation, thereby promoting cellular senescence and SASP production. Transcriptional programs are further fine-tuned by epigenetic mechanisms, including DNA and histone modifications and miRNA regulation. Collectively, these interconnected pathways converge to drive the development and progression of HFpEF. cGAS: cyclic GMP–AMP synthase; cGMP: cyclic guanosine monophosphate; DAMP: damage-associated molecular pattern; DDR: DNA damage response; EAT: epicardial adipose tissue; ER: endoplasmic reticulum; FAO: fatty acid oxidation; HFpEF: heart failure with preserved ejection fraction; IL-1β: interleukin-1β; IL-18: interleukin-18; IRE1α: inositol-requiring enzyme 1α; miRNA: microRNA; mtDNA: mitochondrial DNA; NAD^+^: nicotinamide adenine dinucleotide; NF-κB: nuclear factor kappa-light-chain-enhancer of activated B cells; NLRP3: NLR family pyrin domain-containing 3; NO: nitric oxide; PKG: protein kinase G; PRR: pattern recognition receptor; ROS: reactive oxygen species; SASP: senescence-associated secretory phenotype; STING: stimulator of interferon genes; TGF-β: transforming growth factor beta; UPR: unfolded protein response; XBP1: X-box-binding protein 1.

As discussed above, the preceding sections have summarized the paradigm of systemic and coronary microvascular inflammation, cardiac energy metabolic dysregulation and mitochondrial dysfunction, as well as aging-related cellular senescence and DNA damage in HFpEF. Over the past decade, accumulating evidence has increasingly revealed that these mechanisms engage in extensive bidirectional crosstalk, underscoring the integrative nature of HFpEF pathophysiology. Within this framework, the following sections examine how metabolic comorbidities initiate systemic inflammation, how inflammatory and metabolic perturbations shape cardiac aging, and how diverse metabolites modulate cardiac inflammation and aging in HFpEF. Collectively, these interconnected molecular pathways converge to drive HFpEF pathogenesis (Figure 4).

### Metabolic inflammation in obesity and pressure overload

Metabolic comorbidities are closely associated with systemic inflammation in patients with HFpEF. Elevated IL-6 levels, for example, correlate with greater symptom severity and obesity in this population.^[16]^ Inflammation-related proteins, including TNF receptor 1, urokinase plasminogen activator receptor, insulin-like growth factor-binding protein 7, and GDF-15 have been identified as key links between metabolic burden and diastolic dysfunction.^[87]^ The chronic low-grade inflammation associated with obesity, diabetes, and other metabolic disorders—commonly termed metabolic inflammation or meta-inflammation—has emerged as a central concept in HFpEF pathophysiology.^[11]^ Metabolic diseases sustain systemic inflammation through multiple signaling pathways, whereby metabolic substrates directly modulate gene expression and immune activation. In parallel, inflammation impairs mitochondrial function and disturbs myocardial energy metabolism.^[25,88]^ This bidirectional reinforcement between inflammation and metabolic dysregulation synergistically accelerates the progression of HFpEF.

Dysregulated lipid metabolism and lipotoxicity play pivotal roles in the activation of immune and inflammatory pathways in obesity. Activation of visceral adipose tissue through hypoxia-inducible factor 1α signaling promotes the accumulation of pro-inflammatory long-chain fatty acids, release of inflammatory mediators, and recruitment of immune cells.^[22,89]^ Epicardial adipose tissue (EAT) serves as a key site of immune cell aggregation, forming fat-associated lymphoid clusters, where local inflammation and macrophage infiltration are strongly linked to HFpEF development.^[22,90]^ Expansion of EAT drives adipocytes toward a proinflammatory phenotype, enhancing lipolysis and secretion of cytokines such as leptin, TNF, IL-1β, and IL-6, while reducing adiponectin expression. Adipose-derived chemokines promote macrophage infiltration and polarization toward the proinflammatory M1 phenotype,^[91]^ further amplifying oxidative stress through IL-1β–dependent mitochondrial ROS production.^[88]^ Single-cell analyses in hyperlipidemic HFpEF mouse models demonstrate expansion of inflammatory macrophage subsets driven by lipid-induced endoplasmic reticulum stress, which exacerbates cardiomyocyte hypertrophy, fibrosis, and autophagy impairment.^[24]^ Conversely, overexpression of XBP1 reduces lipid accumulation and myocardial fibrosis by promoting degradation of the transcription factor forkhead box O1, thereby limiting cardiomyocyte lipid deposition.^[92]^

Increased hemodynamic load in hypertension promotes ROS generation and proinflammatory cytokine release within the renal and pulmonary vasculature and induces inflammatory cell infiltration into the myocardium.^[13,93]^ Both hypertension and obesity favor macrophage M1 polarization and myocardial infiltration,^[22,94]^ while hypertensive hearts also exhibit an imbalance in T cell subsets, characterized by a shift toward proinflammatory Th17 cells relative to regulatory T cells.^[22,95]^ Damage-associated molecular patterns (DAMPs) released from injured cardiomyocytes in hypertension—including high-mobility group box 1 (HMGB1), heat shock proteins, DNA fragments, oxidized low-density lipoproteins, and mitochondrial contents—activate pattern recognition receptors (PRRs) such as Toll-like receptors, thereby triggering systemic inflammation.^[22,96]^ The NLRP3 inflammasome is among the most extensively characterized inflammasome sensors in the heart and plays a major role in obesity-related systemic inflammation and insulin resistance.^[97,98]^ DAMPs and cytokines engage PRRs and cytokine receptors to activate NF-κB signaling, inducing the transcription of NLRP3, pro-IL-1β, and pro-IL-18. Upon activation by stimuli such as mitochondrial ROS and long-chain saturated fatty acids, NLRP3 assembles with apoptosis-associated speck-like protein containing a CARD (ASC) and caspase-1, leading to caspase-1 activation and subsequent maturation of IL-1β and IL-18.^[98]^ In addition, increased HMGB1 expression facilitates the formation of neutrophil extracellular traps, which have been shown to mediate diastolic dysfunction in HFpEF mouse models.^[99]^

### Inflammaging and obesity-induced cardiac aging

Aging is associated with elevated circulating proinflammatory mediators, immune dysfunction, and a chronic inflammatory state, even in the absence of overt immunogenic stimuli.^[100]^ This age-related, low-grade inflammation—termed inflammaging—substantially increases the risk of HFpEF in older adults.^[100, 101, 102, 103]^ Inflammaging arises from sustained activation of innate immune pathways and involves multiple converging mechanisms, including cellular senescence, mitochondrial dysfunction, impaired autophagy and mitophagy, inflammasome activation, dysregulated ubiquitin–proteasome activity, DDR activation, and altered microRNA expression.^[100,101]^ The cGAS-STING pathway detects ectopic DNA, including cytosolic mtDNA released from damaged mitochondria, thereby activating type I interferon-mediated innate immune signaling and serving as a key driver of age-associated inflammation.^[104, 105, 106]^ In parallel, age-related alterations in microRNA networks function as epigenetic amplifiers of inflammaging by modulating innate immune signaling, cellular senescence, and metabolic stress responses.^[107,108]^ Notably, inflammaging and metabolic inflammation are mutually reinforcing processes that share overlapping molecular pathways.^[101,102]^ This interaction is particularly relevant to HFpEF, given its strong association with aging and cardiometabolic comorbidities.^[11]^

Aging is also accompanied by metabolic reprogramming characterized by mitochondrial dysfunction, insulin resistance, a shift from glucose oxidation toward anaerobic glycolysis, and an imbalance between fatty acid uptake and utilization, collectively promoting aging phenotypes and increasing susceptibility to age-related diseases.^[109]^ Metabolic comorbidities such as obesity can induce premature cardiac aging.^[110]^ Obesity-related structural and functional cardiac changes—including myocardial hypertrophy, increased stiffness, reduced cardiac reserve, and diastolic dysfunction—closely resemble those observed in aging hearts. Obesity also produces metabolic disturbances that mirror aging-related alterations, and its severity correlates positively with circulating biomarkers of biological aging associated with cardiovascular risk.^[111,112]^ At the molecular level, obesity and cardiovascular aging share several hallmark features, including mitochondrial dysfunction, impaired proteostasis, genomic instability, cellular senescence, neurohormonal signaling alterations, and chronic inflammation.^[103,110]^ In particular, visceral adipose tissue contributes to cardiac aging by secreting profibrotic mediators such as osteopontin, which promotes fibroblast senescence, whereas removal of visceral adipose tissue has been shown to attenuate age-related cardiac fibrosis.^[113]^

### Diverse roles of metabolites in cardiac inflammation and aging

Beyond their role as energy substrates, metabolites function as signaling molecules that regulate inflammation and immune responses.^[11,114,115]^ The ketone body β-hydroxybutyrate suppresses inflammation in HFpEF models by reversing mitochondrial protein hyperacetylation caused by sirtuin 3 downregulation, thereby inhibiting NLRP3 inflammasome activation and reducing IL-1β and IL-18 production.^[116]^ Tricarboxylic acid cycle intermediates also modulate inflammatory signaling and epigenetic regulation. α-ketoglutarate exerts anti-inflammatory effects through histone demethylation, whereas low α-ketoglutarate-to-succinate ratio and fumarate accumulation promote proinflammatory gene expression.^[117,118]^ In classically activated (M1) macrophages, succinate accumulation drives hypoxia-inducible factor 1α activation and IL-1β production.^[114,115,119]^ Although well established, the contribution of these immunometabolic pathways to HFpEF remains incompletely defined.

Nicotinamide adenine dinucleotide (NAD^+^) is a central metabolic cofactor and signaling molecule that serves as a substrate for enzymes involved in DDR, epigenetic regulation, post-translational modifications, and metabolic adaptation to nutritional states.^[120]^ NAD^+^ and its reduced form, NADH, regulate cellular redox balance, bioenergetics, substrate utilization, and mitochondrial biogenesis and dynamics. Maintenance of NAD^+^ homeostasis is essential for normal cardiac metabolism and function, and reduced NAD^+^ levels have been reported in patients with HFpEF.^[121,122]^ Aging-associated mitochondrial dysfunction is characterized by declines in NAD^+^ levels and the NAD^+^/NADH ratio, which impair mitochondrial respiration and accelerate cardiac aging. Mitochondrial NAD^+^ depletion disrupts mitophagy and promotes release of mtDNA into the cytosol, thereby amplifying type I interferon responses through activation of the cGAS-STING pathway.^[106]^ Impaired autophagy, a shared hallmark of aging and HFpEF,^[33,110,123]^ can be restored by NAD^+^ repletion through suppression of insulin-like growth factor 1 signaling, leading to improvement of HFpEF phenotypes.^[123,124]^

During cardiac metabolic remodeling, multiple metabolite-mediated histone and non-histone acylations, including acetylation, lactylation, crotonylation, β-hydroxybutyrylation, succinylation, and O-GlcNAcylation, have been implicated in the regulation of cardiac hypertrophy, aging, and HF.^[125, 126, 127]^ These metabolite-sensitive modifications dynamically respond to nutrient excess, redox imbalance, and hypoxic stress, thereby modulating gene programs associated with cardiac pathology. However, their specific contributions to HFpEF pathogenesis remain incompletely understood. Looking forward, integrative multi-omics approaches, particularly metabolomic profiling, may identify HFpEF-relevant metabolite signatures linked to cardiac aging and disease progression, thus informing mechanism-guided therapeutic development.^[128]^

## Treatment strategies targeting metabolism, inflammation, and aging

**Figure 5:**
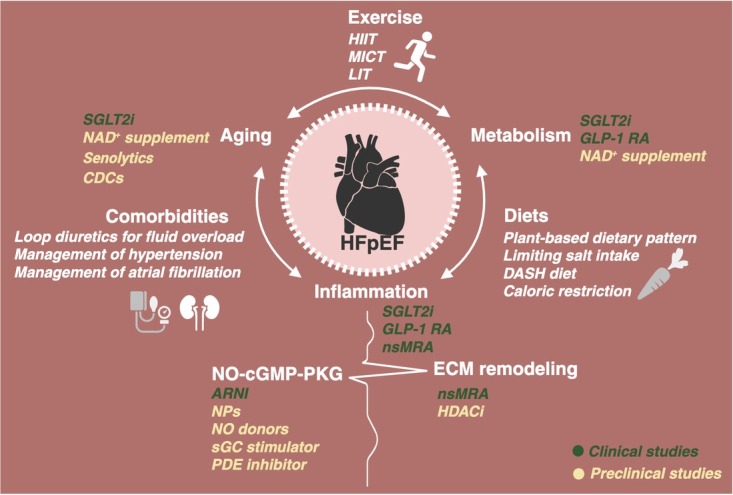
Lifestyle interventions, pharmacologic therapies, and preclinical targets for HFpEF treatment. Lifestyle modifications—including dietary optimization, exercise training, and management of cardiometabolic comorbidities—are fundamental to improving quality of life and clinical outcomes in HFpEF. Several pharmacologic agents, such as ARNIs, SGLT2 inhibitors, GLP-1 RAs, and nsMRAs, improve symptoms and reduce hospitalizations. Emerging preclinical approaches, including NAD^+^ supplementation, modulation of the NO–cGMP–PKG pathway, HDAC inhibition, and anti-aging strategies, target key mechanisms involving inflammation, metabolism, and aging. ARNI: angiotensin receptor–neprilysin inhibitor; CDC: cardiosphere-derived cell; cGMP: cyclic guanosine monophosphate; DASH: Dietary Approaches to Stop Hypertension; GLP-1 RA: glucagon-like peptide-1 receptor agonist; HDAC: histone deacetylase; HFpEF: heart failure with preserved ejection fraction; HIIT: high-intensity interval training; LIT: low-intensity training; MICT: moderate-intensity continuous training; NAD^+^: nicotinamide adenine dinucleotide; NO: nitric oxide; NP: natriuretic peptide; nsMRA: non-steroidal mineralocorticoid receptor antagonist; PDE: phosphodiesterase; PKG: protein kinase G; sGC: soluble guanylate cyclase; SGLT2i: sodium–glucose cotransporter 2 inhibitor.

Although HFpEF remains a major clinical challenge, emerging therapeutic strategies increasingly encompass lifestyle interventions, clinically validated pharmacotherapies, and promising preclinical agents (Figure 5). Many of these approaches converge mechanistically on metabolic remodeling, inflammatory regulation, and aging-related pathways (Table 1). Notably, despite improvements in symptoms and reductions in hospitalization achieved with several approved therapies, none has yet demonstrated a consistent reduction in cardiovascular or all-cause mortality in HFpEF. This highlights the biological heterogeneity and therapeutic refractoriness of the syndrome and emphasizes the need for deeper mechanistic insights.

**Table 1:** Current and emerging pharmacotherapies for HFpEF targeting the inflammation-metabolism-aging axis

Drugs	Primary Targeted Mechanisms	References
Systemic and microvascular inflammation		
SGLT2i	Reduce inflammatory and oxidative stress in HFpEF, improve the NO-sGC-cGMP cascade, alleviate myocardial fibrosis.	[142,143]
GLP-1 RAs	Suppress systemic and endothelial inflammation, reduce myocardial fibrosis.	[151,165,166]
nsMRAs	Inhibit excessive activation of mineralocorticoid receptor and RAAS signaling, exert antifibrotic and anti-inflammatory effects.	[153,154]
Metabolism and metabolic-inflammation		
SGLT2i	Improve glycemic control, reduce body weight and blood pressure; enhance cardiac energetics, induce ketogenesis and autophagy; reduce epicardial adipose tissue accumulation, inhibit NLRP3 inflammasome activation.	[140,141,167,168,169,170]
GLP-1 RAs	Improve glucose homeostasis, blood pressure, reduce appetite, adiposity and inflammation.	[170,171]
NAD^+^ Precursors	Enhance myocardial bioenergetics and metabolic flux, improve mitochondrial function and redox balance, restore autophagy and mitophagy.	
Aging and inflammaging		
SGLT2i	Enhance clearance of senescent cells via immunosurveillance.	[144]
NAD^+^ Precursors	Enhance myocardial bioenergetics and mitochondrial function during aging, activate sirtuins to counteract senescence.	[122,172]
Senolytics	Clearance of senescent cells, attenuate inflammation, cardiac fibrosis and endothelial rarefaction.	[86,161]

### Lifestyle interventions

Lifestyle interventions, including optimization of dietary patterns, increased physical activity, weight loss, and improved control of cardiovascular risk factors, are fundamental to HFpEF management.^[129, 130, 131]^ Dietary modification plays a key role; adherence to plant-based dietary patterns is inversely associated with incident HF, whereas Southern dietary patterns show a positive association with HF risk.^[130,132]^ Sodium restriction alleviates congestion and edema and improves quality of life and clinical outcomes. In patients with hypertensive HFpEF, the sodium-restricted Dietary Approaches to Stop Hypertension (DASH) diet is associated with improvements in diastolic function, arterial elasticity, and ventricular–arterial coupling.^[133]^ Calorie restriction exerts favorable effects on cardiorespiratory fitness, insulin sensitivity, glucose metabolism, and lipid profiles, as well as reductions in body weight, inflammatory burden, and oxidative stress.^[129]^ Intermittent fasting has emerged as a promising strategy to improve cardiometabolic risk factors and cardiovascular outcomes, potentially through circadian alignment and modulation of insulin sensitivity, inflammation, and lipid metabolism.^[134]^ Exercise interventions, including high-intensity interval training, moderate-intensity continuous training, and low-intensity training, consistently improve peak oxygen consumption in patients with HFpEF.^[131]^ Mechanistically, exercise restores skeletal-muscle fatty-acid and branched-chain amino-acid oxidation, thereby improving exercise capacity in a mouse model of cardiometabolic HFpEF.^[135]^ In addition, exercise training protects against cardiac aging and attenuates aging-related pathways in aged mouse models of HFpEF.^[136,137]^ Overall, lifestyle interventions enhance quality of life, exercise capacity, and prognosis, although long-term adherence and the precise mechanisms underlying their benefits require further investigation.

### Clinically proven effective medications for HFpEF

#### SGLT2 inhibitors

SGLT2 inhibitors have emerged as first-line therapy for HFpEF.^[4]^ These agents improve glycemic control, reduce body weight and blood pressure, and confer cardiovascular benefits.^[138]^ Mechanistically, SGLT2 inhibitors modulate metabolic state, enhance autophagic flux, promote ketogenesis and erythropoiesis, and counteract insulin resistance.^[139, 140, 141]^ In addition, SGLT2 inhibitors reduce EAT accumulation, inhibit NLRP3 inflammasome activation, improve systemic metabolic and inflammatory profiles, and reduce myocardial fibrosis.^[24,139,142]^ Consistent with the microvascular inflammation paradigm, empagliflozin reduces myocardial ICAM-1, vascular cell adhesion molecule 1, TNF, and IL-6 expression, attenuates cardiomyocyte oxidative stress, and improves myocardial compliance by enhancing the NO–sGC–cGMP cascade and PKG1α activity.^[143]^ Besides, SGLT2 inhibitors also promote clearance of senescent cells through enhanced immunosurveillance.^[144]^ Beyond cardiac effects, SGLT2 inhibitors slow renal function decline by reducing transglomerular pressure, modulating tubuloglomerular feedback, and constricting afferent arterioles, with synergistic benefits alongside renin–angiotensin–aldosterone system (RAAS) inhibition. These combined cardiovascular and renal effects underpin their clinical efficacy in HFpEF.^[145]^

#### Glucagon-Like peptide-1 receptor agonists (GLP-1 RAs)

GLP-1 RAs improve glucose homeostasis through actions on pancreatic islet cells, regulation of gastric emptying, and central nervous system pathways, leading to reduced appetite, body weight, blood pressure, adiposity, and systemic inflammation.^[146]^ The STEP-HFpEF trial demonstrated significant clinical benefits of semaglutide in patients with obesity-related HFpEF, including symptom improvement, enhanced exercise tolerance, and reduced inflammatory burden.^[147]^ Treatment with tirzepatide, a long-acting dual agonist of glucose-dependent insulinotropic polypeptide and GLP-1 receptors, was also associated with lower cardiovascular risk in obesity-related HFpEF; however, these benefits were driven primarily by reductions in worsening HF events rather than cardiovascular mortality.^[148]^ In preclinical studies, liraglutide improved cardiac metabolism, attenuated ventricular hypertrophy and fibrosis, preserved cardiac structure and function, and reduced circulating natriuretic peptide levels in a multi-hit mouse model of HFpEF.^[149]^ Liraglutide also increased endothelial eNOS expression, reduced ICAM-1 levels, and enhanced PKG signaling in cardiomyocytes.^[150,151]^ As interest in GLP-1 RAs continues to grow, further studies are needed to confirm their efficacy across distinct HFpEF subgroups, clarify effects on hard clinical endpoints, and elucidate underlying molecular mechanisms.

#### Nonsteroidal mineralocorticoid receptor antagonists

Finerenone is a nonsteroidal mineralocorticoid receptor antagonist with greater receptor-binding affinity and selectivity, a shorter plasma half-life, and a lower risk of hyperkalemia than spironolactone and eplerenone.^[152]^ By inhibiting excessive activation of mineralocorticoid receptor and RAAS signaling, finerenone exerts antihypertrophic, antifibrotic, and anti-inflammatory effects, conferring clinically meaningful cardiorenal benefits.^[153, 154, 155]^ In the FINEARTS-HF trial,^[156]^ finerenone reduced the primary composite outcome of total worsening HF events and cardiovascular death by 16% in patients with HFpEF and mildly reduced ejection fraction, while also improving quality of life. The reduction was largely driven by an 18% decrease in worsening HF events, with no significant effect on cardiovascular mortality. Subgroup analyses showed similar treatment effects regardless of concomitant SGLT2 inhibitor use. The ongoing CONFIRMATION-HF trial (NCT06024746) is evaluating the efficacy and safety of combined finerenone and SGLT2 inhibitor therapy compared with standard care in hospitalized patients across the full spectrum of ejection fraction.

### ***Potential preclinical therapeutic targets*** NAD^+^

Preclinical studies demonstrate that supplementation with NAD^+^ precursors, such as nicotinamide and nicotinamide riboside, improves diastolic dysfunction in aging and cardiometabolic HFpEF models.^[121,122]^ Activation of nicotinamide phosphoribosyltransferase (NAMPT), the rate-limiting enzyme in the NAD^+^ salvage pathway, confers similar benefits. Mechanistically, nicotinamide enhances myocardial bioenergetics by promoting weight loss, shifting substrate metabolism utilization toward fatty acid β-oxidation, and deacetylating key regulators of diastolic function, thereby improving cardiomyocyte passive tension and calcium handling.^[122]^ Additionally, nicotinamide restores autophagy and mitophagy in obesity-related HFpEF models.^[123,124]^ Collectively, these findings identify NAD^+^ augmentation as a promising therapeutic strategy, warranting further clinical investigation.

#### Targeting the NO–cGMP–PKG pathway

Although the NO–cGMP–PKG pathway is central to HFpEF, most clinical trials targeting this axis—including NO donors, natriuretic peptides, sGC stimulators, and phosphodiesterase (PDE) inhibitors—have shown neutral or inconsistent results.^[36]^ Proposed explanations include nitrosative stress,^[36,46]^ and compartmentalization of cGMP signaling within cardiomyocytes.^[13]^ cGMP generated *via* the NO–sGC–PDE5 pathway is restricted to the Z-disc and subject to feedback inhibition, limiting phosphorylation of myofilament proteins such as titin.^[13]^ In contrast, natriuretic peptide–particulate guanylyl cyclase–PDE9 signaling localizes closer to titin, contributing to benefits observed with angiotensin receptor–neprilysin inhibitors.^[157]^ Vasodilator-induced hypotension further limits NO-based therapies. Ongoing trials evaluating more targeted modulation of this pathway may clarify therapeutic potential.

#### HDAC inhibitors

HDAC inhibitors, currently approved for several malignancies, demonstrate cardioprotective effects in HFpEF models by reducing fibrosis, inhibiting collagen synthesis, and reversing ventricular hypertrophy.^[158]^ These agents improve diastolic function by enhancing myofibrillar relaxation ^[159]^ and attenuating pathological ECM remodeling.^[57]^ However, adverse effects, including arrhythmias, atherosclerosis, and vascular calcification,^[160]^ limit clinical applicability. More selective approaches to HDAC modulation are therefore required.

#### Anti-Aging and cardiac regeneration strategies

Anti-aging strategies include senolytics, senostatics, and SASP inhibitors.^[78]^ In preclinical models, senolytics reduce cardiomyocyte hypertrophy and fibrosis, along with attenuating systemic inflammation and endothelial rarefaction.^[86,161]^ Furthermore, improved diastolic relaxation was demonstrated in a human heart-on-a-chip model with dasatinib and quercetin.^[162]^ Nevertheless, their long-term safety and efficacy remain uncertain. Cardiac regeneration approaches, such as cardiosphere-derived cell therapy, improve diastolic function in experimental models *via* antifibrotic and anti-inflammatory mechanisms,^[163]^ partly mediated by inhibition of protein kinase C β.^[164]^ While promising, these strategies remain experimental and require further validation.

## Summary and future perspectives

Over the past decade, comorbidity-driven systemic inflammation has emerged as a central framework for understanding HFpEF pathogenesis. This concept has expanded beyond a purely microvascular model. Growing evidence indicates that cardiomyocytes exhibit intrinsic dysfunction, including altered titin phosphorylation, mitochondrial impairment, oxidative and nitrosative stress, and maladaptive metabolic reprogramming. Importantly, these changes may occur independently of endothelial injury, suggesting that cardiomyocyte stress can function as an initiating or parallel driver of disease rather than solely a downstream consequence. Although inflammation remains a core feature, its predominant sources, whether endothelial cells, cardiomyocytes, immune cells, or adipose tissue, may vary across HFpEF subtypes, representing a critical area for future investigation.

Recent studies highlight the interplay among immune activation, metabolic dysregulation, and aging, now conceptualized within the emerging inflammation–metabolism–aging framework that underpins HFpEF pathophysiology. As understanding of these interconnected mechanisms expands, novel therapeutic targets are being identified, creating new opportunities for treatment development. The field is increasingly moving away from a one-pathway-fits-all approach toward a precision medicine paradigm that acknowledges the marked heterogeneity of HFpEF phenotypes. In this context, biomarker-guided patient stratification constitutes a pivotal step toward precision medicine in HFpEF. Given that inflammation, metabolic dysregulation, and aging-related pathways differentially dominate disease progression among patients, contributing to the intrinsic heterogeneity of HFpEF, specific biomarkers could be employed to identify the predominant pathophysiological phenotype in each patient. This stratification would permit rational allocation of targeted therapies, such as anti-inflammatory, metabolic, or senescence-modulating agents, to individuals most likely to benefit, thereby shifting from phenotype-based classification to mechanism-based treatment.

Several key questions remain to be addressed to advance mechanistic insight and therapeutic development in HFpEF: (1) Molecular targets: Determining actionable molecular pathways and targets that regulate inflammation, energy metabolism, aging, and their mechanistic convergence in HFpEF; (2) Cellular interactions: Elucidating the roles and interrelationships of cardiomyocytes, endothelial cells, fibroblasts, macrophages, and other cell types at single-cell resolution; (3) Inter-organ crosstalk: Defining systemic interactions among the heart, kidney, liver, brain, and skeletal muscle to more accurately characterize HFpEF as a multisystem disorder; (4) Clinical heterogeneity: Identifying molecular determinants underlying phenotypic variability, including sex-related differences and distinct HFpEF subgroups; (5) Divergence from HFrEF: Clarifying the mechanisms that differentiate HFpEF from HFrEF across the heart-failure continuum; (6) Translational advances: Accelerating the translation of mechanistic discoveries into precision biomarkers and effective therapies for HFpEF.

Given its complexity, multisystem involvement, and substantial clinical heterogeneity, HFpEF necessitates a multidisciplinary research approach. Integration of basic, translational, and clinical investigations will be essential to advance understanding and to develop effective, personalized therapeutic strategies for this challenging condition.

## References

[1] Heidenreich PA, Bozkurt B, Aguilar D, Allen LA, Byun JJ, Colvin MM (2022). AHA/ACC/HFSA Guideline for the Management of Heart Failure: Executive Summary: A Report of the American College of Cardiology/American Heart Association Joint Committee on Clinical Practice Guidelines. J Am Coll Cardiol.

[2] Hamo CE, DeJong C, Hartshorne-Evans N, Lund LH, Shah SJ, Solomon S (2024). Heart failure with preserved ejection fraction. Nat Rev Dis Primers.

[3] Khan MS, Shahid I, Bennis A, Rakisheva A, Metra M, Butler J (2024). Global epidemiology of heart failure. Nat Rev Cardiol.

[4] Redfield MM, Borlaug BA (2023). Heart Failure With Preserved Ejection Fraction: A Review. Jama.

[5] Kondo T, Henderson AD, Docherty KF, Jhund PS, Vaduganathan M, Solomon SD (2024). Why Have We Not Been Able to Demonstrate Reduced Mortality in Patients With HFmrEF/HFpEF?. J Am Coll Cardiol.

[6] Peters AE, Tromp J, Shah SJ, Lam CSP, Lewis GD, Borlaug BA (2023). Phenomapping in heart failure with preserved ejection fraction: insights, limitations, and future directions. Cardiovasc Res.

[7] Schiattarella GG, Alcaide P, Condorelli G, Gillette TG, Heymans S, Jones EAV (2022). Immunometabolic Mechanisms of Heart Failure with Preserved Ejection Fraction. Nat Cardiovasc Res.

[8] Omote K, Verbrugge FH, Borlaug BA (2022). Heart Failure with Preserved Ejection Fraction: Mechanisms and Treatment Strategies. Annu Rev Med.

[9] Paulus WJ, Tschöpe C (2013). A novel paradigm for heart failure with preserved ejection fraction: comorbidities drive myocardial dysfunction and remodeling through coronary microvascular endothelial inflammation. J Am Coll Cardiol.

[10] Mishra S, Kass DA (2021). Cellular and molecular pathobiology of heart failure with preserved ejection fraction. Nat Rev Cardiol.

[11] Schiattarella GG, Rodolico D, Hill JA (2021). Metabolic inflammation in heart failure with preserved ejection fraction. Cardiovasc Res.

[12] Schiattarella GG, Sequeira V, Ameri P (2021). Distinctive patterns of inflammation across the heart failure syndrome. Heart Fail Rev.

[13] Paulus WJ, Zile MR (2021). From Systemic Inflammation to Myocardial Fibrosis: The Heart Failure With Preserved Ejection Fraction Paradigm Revisited. Circ Res.

[14] Pugliese NR, Pellicori P, Filidei F, De Biase N, Maffia P, Guzik TJ (2023). Inflammatory pathways in heart failure with preserved left ventricular ejection fraction: implications for future interventions. Cardiovasc Res.

[15] Mesquita T, Lin YN, Ibrahim A (2021). Chronic low-grade inflammation in heart failure with preserved ejection fraction. Aging Cell.

[16] Alogna A, Koepp KE, Sabbah M, Espindola Netto JM, Jensen MD, Kirkland JL (2023). Interleukin-6 in Patients With Heart Failure and Preserved Ejection Fraction. JACC Heart Fail.

[17] Hage C, Michaëlsson E, Linde C, Donal E, Daubert JC, Gan LM (2017). Inflammatory Biomarkers Predict Heart Failure Severity and Prognosis in Patients With Heart Failure With Preserved Ejection Fraction: A Holistic Proteomic Approach. Circ Cardiovasc Genet.

[18] Koller L, Kleber M, Goliasch G, Sulzgruber P, Scharnagl H, Silbernagel G (2014). C-reactive protein predicts mortality in patients referred for coronary angiography and symptoms of heart failure with preserved ejection fraction. Eur J Heart Fail.

[19] Tromp J, Westenbrink BD, Ouwerkerk W, van Veldhuisen DJ, Samani NJ, Ponikowski P (2018). Identifying Pathophysiological Mechanisms in Heart Failure With Reduced Versus Preserved Ejection Fraction. J Am Coll Cardiol.

[20] Hahn VS, Yanek LR, Vaishnav J, Ying W, Vaidya D, Lee YZJ (2020). Endomyocardial Biopsy Characterization of Heart Failure With Preserved Ejection Fraction and Prevalence of Cardiac Amyloidosis. JACC Heart Fail.

[21] Franssen C, Chen S, Unger A, Korkmaz HI, De Keulenaer GW, Tschöpe C (2016). Myocardial Microvascular Inflammatory Endothelial Activation in Heart Failure With Preserved Ejection Fraction. JACC Heart Fail.

[22] Daou D, Gillette TG, Hill JA (2023). Inflammatory Mechanisms in Heart Failure with Preserved Ejection Fraction. Physiology (Bethesda).

[23] Venkatesan T, Toumpourleka M, Niewiadomska M, Farhat K, Morris L, Elkholey K (2025). Vagal Stimulation Rescues HFpEF by Altering Cardiac Resident Macrophage Function. Circ Res.

[24] Panico C, Felicetta A, Kunderfranco P, Cremonesi M, Salvarani N, Carullo P (2024). Single-Cell RNA Sequencing Reveals Metabolic Stress-Dependent Activation of Cardiac Macrophages in a Model of Dyslipidemia-Induced Diastolic Dysfunction. Circulation.

[25] Aimo A, Castiglione V, Borrelli C, Saccaro LF, Franzini M, Masi S (2020). Oxidative stress and inflammation in the evolution of heart failure: From pathophysiology to therapeutic strategies. Eur J Prev Cardiol.

[26] Wang D, Yu X, Gao K, Li F, Li X, Pu H (2024). Sweroside alleviates pres-CaMKⅡsure overload-induced heart failure through targeting δ to inhibit ROS-mediated NF-κB/NLRP3 in cardiomyocytes. Redox Biol.

[27] Shah SJ, Lam CSP, Svedlund S, Saraste A, Hage C, Tan RS (2018). Prevalence and correlates of coronary microvascular dysfunction in heart failure with preserved ejection fraction: PROMIS-HFpEF. Eur Heart J.

[28] Camici PG, Tschöpe C, Di Carli MF, Rimoldi O, Van Linthout S (2020). Coronary microvascular dysfunction in hypertrophy and heart failure. Cardiovasc Res.

[29] Sinha A, Rahman H, Webb A, Shah AM, Perera D (2021). Untangling the pathophysiologic link between coronary microvascular dysfunction and heart failure with preserved ejection fraction. Eur Heart J.

[30] Yang JH, Obokata M, Reddy YNV, Redfield MM, Lerman A, Borlaug BA (2020). Endothelium-dependent and independent coronary microvascular dysfunction in patients with heart failure with preserved ejection fraction. Eur J Heart Fail.

[31] Gevaert AB, Shakeri H, Leloup AJ, Van Hove CE, De Meyer GRY, Vrints CJ (2017). Endothelial Senescence Contributes to Heart Failure With Preserved Ejection Fraction in an Aging Mouse Model. Circ Heart Fail.

[32] Salvador AM, Nevers T, Velázquez F, Aronovitz M, Wang B, Abadía Molina A (2016). Intercellular Adhesion Molecule 1 Regulates Left Ventricular Leukocyte Infiltration, Cardiac Remodeling, and Function in Pressure Overload-Induced Heart Failure. J Am Heart Assoc.

[33] Hahn VS, Knutsdottir H, Luo X, Bedi K, Margulies KB, Haldar SM (2021). Myocardial Gene Expression Signatures in Human Heart Failure With Preserved Ejection Fraction. Circulation.

[34] Horton WB, Barrett EJ (2021). Microvascular Dysfunction in Diabetes Mellitus and Cardiometabolic Disease. Endocr Rev.

[35] Li G, Zhao H, Cheng Z, Liu J, Li G, Guo Y (2025). Single-cell transcriptomic profiling of heart reveals ANGPTL4 linking fibroblasts and angiogenesis in heart failure with preserved ejection fraction. J Adv Res.

[36] Cai Z, Wu C, Xu Y, Cai J, Zhao M, Zu L (2023). The NO-cGMP-PKG Axis in HFpEF: From Pathological Mechanisms to Potential Therapies. Aging Dis.

[37] Paulus WJ (2020). Unfolding Discoveries in Heart Failure. N Engl J Med.

[38] van Heerebeek L, Hamdani N, Falcão-Pires I, Leite-Moreira AF, Begieneman MP, Bronzwaer JG (2012). Low myocardial protein kinase G activity in heart failure with preserved ejection fraction. Circulation.

[39] Loescher CM, Hobbach AJ, Linke WA (2022). Titin (TTN): from molecule to modifications, mechanics, and medical significance. Cardiovasc Res.

[40] Kötter S, Gout L, Von Frieling-Salewsky M, Müller AE, Helling S, Marcus K (2013). Differential changes in titin domain phosphorylation increase myofilament stiffness in failing human hearts. Cardiovasc Res.

[41] Zile MR, Baicu CF, Ikonomidis JS, Stroud RE, Nietert PJ, Bradshaw AD (2015). Myocardial stiffness in patients with heart failure and a preserved ejection fraction: contributions of collagen and titin. Circulation.

[42] Hamdani N, Bishu KG, von Frieling-Salewsky M, Redfield MM, Linke WA (2013). Deranged myofilament phosphorylation and function in experimental heart failure with preserved ejection fraction. Cardiovasc Res.

[43] Loescher CM, Breitkreuz M, Li Y, Nickel A, Unger A, Dietl A (2020). Regulation of titin-based cardiac stiffness by unfolded domain oxidation (UnDOx). Proc Natl Acad Sci U S A.

[44] Mollace R, Scarano F, Bava I, Carresi C, Maiuolo J, Tavernese A (2023). Modulation of the nitric oxide/cGMP pathway in cardiac contraction and relaxation: Potential role in heart failure treatment. Pharmacol Res.

[45] Shah SA, Reagan CE, Bresticker JE, Wolpe AG, Good ME, Macal EH (2023). Obesity-Induced Coronary Microvascular Disease Is Prevented by iNOS Deletion and Reversed by iNOS Inhibition. JACC Basic Transl Sci.

[46] Schiattarella GG, Altamirano F, Tong D, French KM, Villalobos E, Kim SY (2019). Nitrosative stress drives heart failure with preserved ejection fraction. Nature.

[47] Guo Y, Wen J, He A, Qu C, Peng Y, Luo S (2023). iNOS contributes to heart failure with preserved ejection fraction through mitochondrial dysfunction and Akt S-nitrosylation. J Adv Res.

[48] Smolgovsky S, Bayer AL, Kaur K, Sanders E, Aronovitz M, Filipp ME (2023). Impaired T cell IRE1α/XBP1 signaling directs inflammation in experimental heart failure with preserved ejection fraction. J Clin Invest.

[49] Kuwahara F, Kai H, Tokuda K, Kai M, Takeshita A, Egashira K (2002). Transforming growth factor-beta function blocking prevents myocardial fibrosis and diastolic dysfunction in pressure-overloaded rats. Circulation.

[50] Westermann D, Lindner D, Kasner M, Zietsch C, Savvatis K, Escher F (2011). Cardiac inflammation contributes to changes in the extracellular matrix in patients with heart failure and normal ejection fraction. Circ Heart Fail.

[51] Russo I, Cavalera M, Huang S, Su Y, Hanna A, Chen B (2019). Protective Effects of Activated Myofibroblasts in the Pressure-Overloaded Myocardium Are Mediated Through Smad-Dependent Activation of a Matrix-Preserving Program. Circ Res.

[52] Alvandi Z, Bischoff J (2021). Endothelial-Mesenchymal Transition in Cardiovascular Disease. Arterioscler Thromb Vasc Biol.

[53] Hulsmans M, Sager HB, Roh JD, Valero-Muñoz M, Houstis NE, Iwamoto Y (2018). Cardiac macrophages promote diastolic dysfunction. J Exp Med.

[54] Abe H, Tanada Y, Omiya S, Podaru MN, Murakawa T, Ito J (2021). NF-κB activation in cardiac fibroblasts results in the recruitment of inflammatory Ly6C(hi) monocytes in pressure-overloaded hearts. Sci Signal.

[55] Ngwenyama N, Kaur K, Bugg D, Theall B, Aronovitz M, Berland R (2022). Antigen presentation by cardiac fibroblasts promotes cardiac dysfunction. Nat Cardiovasc Res.

[56] Ye B, Bradshaw AD, Abrahante JE, Dragon JA, Häußler TN, Bell SP (2023). Left Ventricular Gene Expression in Heart Failure With Preserved Ejection Fraction-Profibrotic and Proinflammatory Pathways and Genes. Circ Heart Fail.

[57] Travers JG, Wennersten SA, Peña B, Bagchi RA, Smith HE, Hirsch RA (2021). HDAC Inhibition Reverses Preexisting Diastolic Dysfunction and Blocks Covert Extracellular Matrix Remodeling. Circulation.

[58] Yamada Y, Sadahiro T, Nakano K, Honda S, Abe Y, Akiyama T (2025). Cardiac Reprogramming and Gata4 Overexpression Reduce Fibrosis and Improve Diastolic Dysfunction in Heart Failure With Preserved Ejection Fraction. Circulation.

[59] Lopaschuk GD, Karwi QG, Tian R, Wende AR, Abel ED (2021). Cardiac Energy Metabolism in Heart Failure. Circ Res.

[60] Ritterhoff J, Tian R (2023). Metabolic mechanisms in physiological and pathological cardiac hypertrophy: new paradigms and challenges. Nat Rev Cardiol.

[61] Capone F, Sotomayor-Flores C, Bode D, Wang R, Rodolico D, Strocchi S (2023). Cardiac metabolism in HFpEF: from fuel to signalling. Cardiovasc Res.

[62] Wang R, Schiattarella GG (2024). Tackling metabolic defects in HFpEF. Eur Heart J.

[63] Sun Q, Güven B, Wagg CS, Almeida de Oliveira A, Silver H, Zhang L (2024). Mitochondrial fatty acid oxidation is the major source of cardiac adenosine triphosphate production in heart failure with preserved ejection fraction. Cardiovasc Res.

[64] Meddeb M, Koleini N, Binek A, Keykhaei M, Darehgazani R, Kwon S (2024). Myocardial ultrastructure of human heart failure with preserved ejection fraction. Nat Cardiovasc Res.

[65] Hahn VS, Petucci C, Kim MS, Bedi KC Jr, Wang H, Mishra S (2023). Myocardial Metabolomics of Human Heart Failure With Preserved Ejection Fraction. Circulation.

[66] Koleini N, Meddeb M, Zhao L, Keykhaei M, Kwon S, Farshidfar F (2024). Landscape of glycolytic metabolites and their regulating proteins in myocardium from human heart failure with preserved ejection fraction. Eur J Heart Fail.

[67] Leggat J, Bidault G, Vidal-Puig A (2021). Lipotoxicity: a driver of heart failure with preserved ejection fraction?. Clin Sci (Lond).

[68] Fillmore N, Levasseur JL, Fukushima A, Wagg CS, Wang W, Dyck JRB (2018). Uncoupling of glycolysis from glucose oxidation accompanies the development of heart failure with preserved ejection fraction. Mol Med.

[69] Ritchie RH, Abel ED (2020). Basic Mechanisms of Diabetic Heart Disease. Circ Res.

[70] Martinez CS, Zheng A, Xiao Q (2024). Mitochondrial Reactive Oxygen Species Dysregulation in Heart Failure with Preserved Ejection Fraction: A Fraction of the Whole. Antioxidants (Basel).

[71] Kumar AA, Kelly DP, Chirinos JA (2019). Mitochondrial Dysfunction in Heart Failure With Preserved Ejection Fraction. Circulation.

[72] Yoshii A, McMillen TS, Wang Y, Zhou B, Chen H, Banerjee D (2024). Blunted Cardiac Mitophagy in Response to Metabolic Stress Contributes to HFpEF. Circ Res.

[73] Zhou H, Wang X, Xu T, Gan D, Ma Z, Zhang H (2025). PINK1-mediated mitophagy attenuates pathological cardiac hypertrophy by suppressing the mtDNA release-activated cGAS-STING pathway. Cardiovasc Res.

[74] Ho JE, Enserro D, Brouwers FP, Kizer JR, Shah SJ, Psaty BM (2016). Predicting Heart Failure With Preserved and Reduced Ejection Fraction: The International Collaboration on Heart Failure Subtypes. Circ Heart Fail.

[75] Brouwers FP, de Boer RA, van der Harst P, Voors AA, Gansevoort RT, Bakker SJ (2013). Incidence and epidemiology of new onset heart failure with preserved vs. reduced ejection fraction in a community-based cohort: 11-year follow-up of PREVEND. Eur Heart J.

[76] Mehdizadeh M, Aguilar M, Thorin E, Ferbeyre G, Nattel S (2022). The role of cellular senescence in cardiac disease: basic biology and clinical relevance. Nat Rev Cardiol.

[77] Hastings MH, Zhou Q, Wu C, Shabani P, Huang S, Yu X (2025). Cardiac ageing: from hallmarks to therapeutic opportunities. Cardiovasc Res.

[78] Chen MS, Lee RT, Garbern JC (2022). Senescence mechanisms and targets in the heart. Cardiovasc Res.

[79] van Wamel AJ, Ruwhof C, van der Valk-Kokshoom LE, Schrier PI, van der Laarse A (2001). The role of angiotensin II, endothelin-1 and transforming growth factor-beta as autocrine/paracrine mediators of stretch-induced cardiomyocyte hypertrophy. Mol Cell Biochem.

[80] Shi Y, Zhao L, Wang J, Liu X, Bai Y, Cong H (2024). Empagliflozin protects against heart failure with preserved ejection fraction partly by inhibiting the senescence-associated STAT1-STING axis. Cardiovasc Diabetol.

[81] Higo T, Naito AT, Sumida T, Shibamoto M, Okada K, Nomura S (2017). DNA single-strand break-induced DNA damage response causes heart failure. Nat Commun.

[82] Nakada Y, Nhi Nguyen NU, Xiao F, Savla JJ, Lam NT, Abdisalaam S (2019). DNA Damage Response Mediates Pressure Overload-Induced Cardiomyocyte Hypertrophy. Circulation.

[83] Cao Y, Pan C, Wang YC, Zhou Z, Jedian V, Meng Y (2022). Identification of DNA Damage Repair Enzyme Ascc2 as Causal for Heart Failure With Preserved Ejection Fraction. Circulation.

[84] Shay JW, Wright WE (2019). Telomeres and telomerase: three decades of progress. Nat Rev Genet.

[85] Sharifi-Sanjani M, Oyster NM, Tichy ED, Bedi KC, Harel O, Margulies KB (2017). Cardiomyocyte-Specific Telomere Shortening is a Distinct Signature of Heart Failure in Humans. J Am Heart Assoc.

[86] Anderson R, Lagnado A, Maggiorani D, Walaszczyk A, Dookun E, Chapman J (2019). Length-independent telomere damage drives post-mitotic cardiomyocyte senescence. Embo j.

[87] Sanders-van Wijk S, Tromp J, Beussink-Nelson L, Hage C, Svedlund S, Saraste A (2020). Proteomic Evaluation of the Comorbidity-Inflammation Paradigm in Heart Failure With Preserved Ejection Fraction: Results From the PROMIS-HFpEF Study. Circulation.

[88] Liu H, Huang Y, Zhao Y, Kang GJ, Feng F, Wang X (2022). Inflammatory Macrophage Interleukin-1β Mediates High-Fat Diet-Induced Heart Failure With Preserved Ejection Fraction. JACC Basic Transl Sci.

[89] Lee YS, Kim JW, Osborne O, Oh DY, Sasik R, Schenk S (2014). Increased adipocyte O2 consumption triggers HIF-1α, causing inflammation and insulin resistance in obesity. Cell.

[90] van Woerden G, van Veldhuisen DJ, Westenbrink BD, de Boer RA, Rienstra M, Gorter TM (2022). Connecting epicardial adipose tissue and heart failure with preserved ejection fraction: mechanisms, management and modern perspectives. Eur J Heart Fail.

[91] Saltiel AR, Olefsky JM (2017). Inflammatory mechanisms linking obesity and metabolic disease. J Clin Invest.

[92] Schiattarella GG, Altamirano F, Kim SY, Tong D, Ferdous A, Piristine H (2021). Xbp1s-FoxO1 axis governs lipid accumulation and contractile performance in heart failure with preserved ejection fraction. Nat Commun.

[93] O’Brien M, Baicu CF, Van Laer AO, Zhang Y, McDonald LT, LaRue AC (2020). Pressure overload generates a cardiac-specific profile of inflammatory mediators. Am J Physiol Heart Circ Physiol.

[94] Mouton AJ, Li X, Hall ME, Hall JE (2020). Obesity, Hypertension, and Cardiac Dysfunction: Novel Roles of Immunometabolism in Macrophage Activation and Inflammation. Circ Res.

[95] Herrada AA, Contreras FJ, Marini NP, Amador CA, González PA, Cortés CM (2010). Aldosterone promotes autoimmune damage by enhancing Th17-mediated immunity. J Immunol.

[96] Ghigo A, Franco I, Morello F, Hirsch E (2014). Myocyte signalling in leucocyte recruitment to the heart. Cardiovasc Res.

[97] Thorp EB, Filipp M (2025). Contributions of Inflammation to Cardiometabolic Heart Failure with Preserved Ejection Fraction. Annu Rev Pathol.

[98] Li C, Qin D, Hu J, Yang Y, Hu D, Yu B (2022). Inflamed adipose tissue: A culprit underlying obesity and heart failure with preserved ejection fraction. Front Immunol.

[99] Zhang XL, Wang TY, Chen Z, Wang HW, Yin Y, Wang L (2022). HMGB1-Promoted Neutrophil Extracellular Traps Contribute to Cardiac Diastolic Dysfunction in Mice. J Am Heart Assoc.

[100] Ferrucci L, Fabbri E (2018). Inflammageing: chronic inflammation in ageing, cardiovascular disease, and frailty. Nat Rev Cardiol.

[101] Liberale L, Montecucco F, Tardif JC, Libby P, Camici GG (2020). Inflamm-ageing: the role of inflammation in age-dependent cardiovascular disease. Eur Heart J.

[102] Franceschi C, Garagnani P, Parini P, Giuliani C, Santoro A (2018). Inflammaging: a new immune-metabolic viewpoint for age-related diseases. Nat Rev Endocrinol.

[103] Abdellatif M, Rainer PP, Sedej S, Kroemer G (2023). Hallmarks of cardiovascular ageing. Nat Rev Cardiol.

[104] Kim J, Kim HS, Chung JH (2023). Molecular mechanisms of mitochondrial DNA release and activation of the cGAS-STING pathway. Exp Mol Med.

[105] Gulen MF, Samson N, Keller A, Schwabenland M, Liu C, Glück S (2023). cGAS-STING drives ageing-related inflammation and neurodegeneration. Nature.

[106] Lan T, Shang D, Lin L, Wang H, Zou J, Hu M (2026). Mitochondrial NAD(+)-mediated mitophagy alleviates type I interferon response to the cytosolic mitochondrial DNA. Autophagy.

[107] Aranda JF, Ramírez CM, Mittelbrunn M (2025). Inflammageing, a targetable pathway for preventing cardiovascular diseases. Cardiovasc Res.

[108] Hamdani N, Costantino S, Mügge A, Lebeche D, Tschöpe C, Thum T (2021). Leveraging clinical epigenetics in heart failure with preserved ejection fraction: a call for individualized therapies. Eur Heart J.

[109] Xie S, Xu SC, Deng W, Tang Q (2023). Metabolic landscape in cardiac aging: insights into molecular biology and therapeutic implications. Signal Transduct Target Ther.

[110] Ruperez C, Madeo F, de Cabo R, Kroemer G, Abdellatif M (2025). Obesity accelerates cardiovascular ageing. Eur Heart J.

[111] Niemann B, Chen Y, Teschner M, Li L, Silber RE, Rohrbach S (2011). Obesity induces signs of premature cardiac aging in younger patients: the role of mitochondria. J Am Coll Cardiol.

[112] Argentieri MA, Xiao S, Bennett D, Winchester L, Nevado-Holgado AJ, Ghose U (2024). Proteomic aging clock predicts mortality and risk of common age-related diseases in diverse populations. Nat Med.

[113] Sawaki D, Czibik G, Pini M, Ternacle J, Suffee N, Mercedes R (2018). Visceral Adipose Tissue Drives Cardiac Aging Through Modulation of Fibroblast Senescence by Osteopontin Production. Circulation.

[114] Bertero E, Dudek J, Cochain C, Delgobo M, Ramos G, Gerull B (2022). Immuno-metabolic interfaces in cardiac disease and failure. Cardiovasc Res.

[115] Ryan DG, O’Neill LAJ (2020). Krebs Cycle Reborn in Macrophage Immunometabolism. Annu Rev Immunol.

[116] Deng Y, Xie M, Li Q, Xu X, Ou W, Zhang Y (2021). Targeting Mitochondria-Inflammation Circuit by β-Hydroxybutyrate Mitigates HFpEF. Circ Res.

[117] Liu PS, Wang H, Li X, Chao T, Teav T, Christen S (2017). α-ketoglutarate orchestrates macrophage activation through metabolic and epigenetic reprogramming. Nat Immunol.

[118] Arts RJ, Novakovic B, Ter Horst R, Carvalho A, Bekkering S, Lachmandas E (2016). Glutaminolysis and Fumarate Accumulation Integrate Immunometabolic and Epigenetic Programs in Trained Immunity. Cell Metab.

[119] Tannahill GM, Curtis AM, Adamik J, Palsson-McDermott EM, McGettrick AF, Goel G (2013). Succinate is an inflammatory signal that induces IL-1β through HIF-1α. Nature.

[120] Abdellatif M, Sedej S, Kroemer G (2021). NAD (+) Metabolism in Cardiac Health, Aging, and Disease. Circulation.

[121] Tong D, Schiattarella GG, Jiang N, Altamirano F, Szweda PA, Elnwasany A (2021). NAD (+) Repletion Reverses Heart Failure With Preserved Ejection Fraction. Circ Res.

[122] Abdellatif M, Trummer-Herbst V, Koser F, Durand S, Adão R, Vasques-Nóvoa F (2021). Nicotinamide for the treatment of heart failure with preserved ejection fraction. Sci Transl Med.

[123] Abdellatif M, Vasques-Nóvoa F, Trummer-Herbst V, Durand S, Koser F, Islam M (2025). Autophagy is required for the therapeutic effects of the NAD+ precursor nicotinamide in obesity-related heart failure with preserved ejection fraction. Eur Heart J.

[124] Abdellatif M, Vasques-Nóvoa F, Ferreira JP, Sadoshima J, Diwan A, Linke WA (2025). NAD (+) repletion restores cardioprotective autophagy and mitophagy in obesity-associated heart failure by suppressing excessive trophic signaling. Autophagy.

[125] Guo L, Du Y, Li H, He T, Yao L, Yang G (2025). Metabolites-mediated posttranslational modifications in cardiac metabolic remodeling: Implications for disease pathology and therapeutic potential. Metabolism.

[126] An Y, Wang Q, Gao K, Zhang C, Ouyang Y, Li R (2025). Epigenetic Regulation of Aging and its Rejuvenation. MedComm (2020).

[127] Liu YQ, Yang Q, He GW (2025). Post-translational acylation of proteins in cardiac hypertrophy. Nat Rev Cardiol.

[128] Huang H, Chen Y, Xu W, Cao L, Qian K, Bischof E (2025). Decoding aging clocks: New insights from metabolomics. Cell Metab.

[129] Vacca A, Wang R, Nambiar N, Capone F, Farrelly C, Mostafa A (2024). Lifestyle interventions in cardiometabolic HFpEF: dietary and exercise modalities. Heart Fail Rev.

[130] Lara KM, Levitan EB, Gutierrez OM, Shikany JM, Safford MM, Judd SE (2019). Dietary Patterns and Incident Heart Failure in U. S. Adults Without Known Coronary Disease. Journal of the American College of Cardiology.

[131] Walters GWM, Yeo JL, Bilak JM, Pepper C, Gulsin GS, Freeman SC (2024). The Effectiveness of Lifestyle Interventions in Heart Failure With Preserved Ejection Fraction: A Systematic Review and Network Meta-Analysis. J Card Fail.

[132] Fuerlinger A, Stockner A, Sedej S, Abdellatif M (2025). Caloric restriction and its mimetics in heart failure with preserved ejection fraction: mechanisms and therapeutic potential. Cardiovasc Diabetol.

[133] Hummel SL, Seymour EM, Brook RD, Kolias TJ, Sheth SS, Rosenblum HR (2012). Low-sodium dietary approaches to stop hypertension diet reduces blood pressure, arterial stiffness, and oxidative stress in hypertensive heart failure with preserved ejection fraction. Hypertension.

[134] Zhang J, Chen Y, Zhong Y, Wang Y, Huang H, Xu W (2025). Intermittent fasting and cardiovascular health: a circadian rhythm-based approach. Sci Bull (Beijing).

[135] Quiriarte H, Noland RC, Stampley JE, Davis G, Li Z, Cho E (2024). Exercise Therapy Rescues Skeletal Muscle Dysfunction and Exercise Intolerance in Cardiometabolic HFpEF. JACC Basic Transl Sci.

[136] Hastings MH, Zhou Q, Wu C, Shabani P, Huang S, Yu X (2025). Cardiac ageing: from hallmarks to therapeutic opportunities. Cardiovasc Res.

[137] Roh JD, Houstis N, Yu A, Chang B, Yeri A, Li H (2020). Exercise training reverses cardiac aging phenotypes associated with heart failure with preserved ejection fraction in male mice. Aging Cell.

[138] Anker SD, Butler J, Filippatos G, Ferreira JP, Bocchi E, Böhm M (2021). Empagliflozin in Heart Failure with a Preserved Ejection Fraction. N Engl J Med.

[139] Pandey AK, Bhatt DL, Pandey A, Marx N, Cosentino F, Pandey A (2023). Mechanisms of benefits of sodium-glucose cotransporter 2 inhibitors in heart failure with preserved ejection fraction. Eur Heart J.

[140] Santos-Gallego CG, Requena-Ibanez JA, San Antonio R, Ishikawa K, Watanabe S, Picatoste B (2019). Empagliflozin Ameliorates Adverse Left Ventricular Remodeling in Nondiabetic Heart Failure by Enhancing Myocardial Energetics. J Am Coll Cardiol.

[141] Zannad F, Ferreira JP, Butler J, Filippatos G, Januzzi JL, Sumin M (2022). Effect of empagliflozin on circulating proteomics in heart failure: mechanistic insights into the EMPEROR programme. Eur Heart J.

[142] Li C, Zhang J, Xue M, Li X, Han F, Liu X (2019). SGLT2 inhibition with empagliflozin attenuates myocardial oxidative stress and fibrosis in diabetic mice heart. Cardiovasc Diabetol.

[143] Kolijn D, Pabel S, Tian Y, Lodi M, Herwig M, Carrizzo A (2021). Empagliflozin improves endothelial and cardiomyocyte function in human heart failure with preserved ejection fraction via reduced pro-inflammatory-oxidative pathways and protein kinase Galpha oxidation. Cardiovasc Res.

[144] Katsuumi G, Shimizu I, Suda M, Yoshida Y, Furihata T, Joki Y (2024). SGLT2 inhibition eliminates senescent cells and alleviates pathological aging. Nat Aging.

[145] Solomon SD, McMurray JJV, Claggett B, de Boer RA, DeMets D, Hernandez AF (2022). Dapagliflozin in Heart Failure with Mildly Reduced or Preserved Ejection Fraction. N Engl J Med.

[146] Ussher JR, Drucker DJ (2023). Glucagon-like peptide 1 receptor agonists: cardiovascular benefits and mechanisms of action. Nat Rev Cardiol.

[147] Kosiborod MN, Abildstrøm SZ, Borlaug BA, Butler J, Rasmussen S, Davies M (2023). Semaglutide in Patients with Heart Failure with Preserved Ejection Fraction and Obesity. N Engl J Med.

[148] Packer M, Zile MR, Kramer CM, Baum SJ, Litwin SE, Menon V (2025). Tirzepatide for Heart Failure with Preserved Ejection Fraction and Obesity. N Engl J Med.

[149] Withaar C, Meems LMG, Markousis-Mavrogenis G, Boogerd CJ, Sillje HHW, Schouten EM (2021). The effects of liraglutide and dapagliflozin on cardiac function and structure in a multi-hit mouse model of heart failure with preserved ejection fraction. Cardiovasc Res.

[150] Wang TY, Yang Q, Cheng XY, Ding JC, Hu PF (2025). Beyond weight loss: the potential of glucagon-like peptide-1 receptor agonists for treating heart failure with preserved ejection fraction. Heart Fail Rev.

[151] Gaspari T, Liu H, Welungoda I, Hu Y, Widdop RE, Knudsen LB (2011). A GLP-1 receptor agonist liraglutide inhibits endothelial cell dysfunction and vascular adhesion molecule expression in an ApoE-/- mouse model. Diab Vasc Dis Res.

[152] Chen X, Huang M, Chen Y, Xu H, Wu M (2025). Mineralocorticoid receptor antagonists and heart failure with preserved ejection fraction: current understanding and future prospects. Heart Failure Reviews.

[153] Grune J, Beyhoff N, Smeir E, Chudek R, Blumrich A, Ban Z (2018). Selective Mineralocorticoid Receptor Cofactor Modulation as Molecular Basis for Finerenone’s Antifibrotic Activity. Hypertension.

[154] Kolkhof P, Delbeck M, Kretschmer A, Steinke W, Hartmann E, Bärfacker L (2014). Finerenone, a novel selective nonsteroidal mineralocorticoid receptor antagonist protects from rat cardiorenal injury. J Cardiovasc Pharmacol.

[155] Agarwal R, Kolkhof P, Bakris G, Bauersachs J, Haller H, Wada T (2021). Steroidal and non-steroidal mineralocorticoid receptor antagonists in cardiorenal medicine. Eur Heart J.

[156] Solomon SD, McMurray JJV, Vaduganathan M, Claggett B, Jhund PS, Desai AS (2024). Finerenone in Heart Failure with Mildly Reduced or Preserved Ejection Fraction. N Engl J Med.

[157] Solomon SD, McMurray JJV, Anand IS, Ge J, Lam CSP, Maggioni AP (2019). Angiotensin-Neprilysin Inhibition in Heart Failure with Preserved Ejection Fraction. N Engl J Med.

[158] Morales CR, Li DL, Pedrozo Z, May HI, Jiang N, Kyrychenko V (2016). Inhibition of class I histone deacetylases blunts cardiac hypertrophy through TSC2-dependent mTOR repression. Sci Signal.

[159] Wallner M, Eaton DM, Berretta RM, Liesinger L, Schittmayer M, Gindlhuber J (2020). HDAC inhibition improves cardiopulmonary function in a feline model of diastolic dysfunction. Sci Transl Med.

[160] Li P, Ge J, Li H (2020). Lysine acetyltransferases and lysine deacetylases as targets for cardiovascular disease. Nat Rev Cardiol.

[161] Silva ED, Tomé I, Vasques-Nóvoa F, Conceição G, Silva A, Barros AS (2025). Pharmacological clearance of senescent cells reduces inflammation, endothelial damage and cardiac fibrosis in HFpEF. Cardiovasc Res.

[162] Mourad O, Masse S, Subha T, Mirshafiei F, Plakhotnik J, Suthiwanich K (2025). Human heart-on-a-chip model emulates structural and functional characteristics of diastolic dysfunction and reveals a beneficial role for senolytics. Acta Biomater.

[163] Gallet R, de Couto G, Simsolo E, Valle J, Sun B, Liu W (2016). Cardiosphere-derived cells reverse heart failure with preserved ejection fraction (HFpEF) in rats by decreasing fibrosis and inflammation. JACC Basic Transl Sci.

[164] Soetkamp D, Gallet R, Parker SJ, Holewinski R, Venkatraman V, Peck K (2021). Myofilament Phosphorylation in Stem Cell Treated Diastolic Heart Failure. Circ Res.

[165] Verma S, Petrie MC, Borlaug BA, Butler J, Davies MJ, Kitzman DW (2024). Inflammation in Obesity-Related HFpEF: The STEP-HFpEF Program. J Am Coll Cardiol.

[166] Withaar C, Meems LMG, Markousis-Mavrogenis G, Boogerd CJ, Silljé HHW, Schouten EM (2021). The effects of liraglutide and dapagliflozin on cardiac function and structure in a multi-hit mouse model of heart failure with preserved ejection fraction. Cardiovasc Res.

[167] Bolinder J, Ljunggren Ö, Kullberg J, Johansson L, Wilding J, Langkilde AM (2012). Effects of dapagliflozin on body weight, total fat mass, and regional adipose tissue distribution in patients with type 2 diabetes mellitus with inadequate glycemic control on metformin. J Clin Endocrinol Metab.

[168] Baker WL, Buckley LF, Kelly MS, Bucheit JD, Parod ED, Brown R (2017). Effects of Sodium-Glucose Cotransporter 2 Inhibitors on 24-Hour Ambulatory Blood Pressure: A Systematic Review and Meta-Analysis. J Am Heart Assoc.

[169] Kim SR, Lee SG, Kim SH, Kim JH, Choi E, Cho W (2020). SGLT2 inhibition modulates NLRP3 inflammasome activity via ketones and insulin in diabetes with cardiovascular disease. Nat Commun.

[170] Myasoedova VA, Parisi V, Moschetta D, Valerio V, Conte M, Massaiu I (2023). Efficacy of cardiometabolic drugs in reduction of epicardial adipose tissue: a systematic review and meta-analysis. Cardiovasc Diabetol.

[171] Sandsdal RM, Juhl CR, Jensen SBK, Lundgren JR, Janus C, Blond MB (2023). Combination of exercise and GLP-1 receptor agonist treatment reduces severity of metabolic syndrome, abdominal obesity, and inflammation: a randomized controlled trial. Cardiovasc Diabetol.

[172] Walker MA, Tian R (2024). NAD metabolism and heart failure: Mechanisms and therapeutic potentials. J Mol Cell Cardiol.

